# Exercise capacity in a cohort of children with congenital heart disease

**DOI:** 10.1007/s00431-022-04648-9

**Published:** 2022-11-05

**Authors:** Wouter J. van Genuchten, Willem A. Helbing, Arend D. J. Ten Harkel, Zina Fejzic, Irene M. Kuipers MD, Martijn G. Slieker, Jelle P. G. van der Ven, Eric Boersma, Tim Takken, Beatrijs Bartelds

**Affiliations:** 1 Department of Pediatrics, Division of Pediatric Cardiology, Erasmus MC, University Medical Center, Room number Sp2469 attn. Prof. Dr. W.A. Helbing, PO box 2040, 3000 CA Zuid Holland Rotterdam, The Netherlands; 2 Department of Cardiology, Erasmus MC, University Medical Center, Rotterdam, The Netherlands; 3 Department of Pediatric Cardiology, Radboud University Medical Center, Nijmegen, The Netherlands; 4 Department of Pediatric Cardiology, Leiden University Medical Center, Leiden, The Netherlands; 5 Department of Pediatric Cardiology, Amsterdam University Medical Center, Amsterdam, the Netherlands; 6 Netherlands Heart Institute, Wilhelmina Children’s Hospital, University Medical Center Utrecht, Utrecht, the Netherlands; 7 Department of Medical Physiology, Wilhelmina Children’s Hospital, University Medical Center Utrecht, Utrecht, The Netherlands; 8 Department of Pediatric Cardiology, Wilhelmina Children’s Hospital, University Medical Center Utrecht, Utrecht, The Netherlands

**Keywords:** Congenital heart disease, Pediatric cardiology, Cardiopulmonary exercise testing, Exercise tolerance, Peak oxygen uptake

## Abstract

In patients with congenital heart disease (CHD), reduced exercise capacity can be a predictor for late complications and may be used to guide interventions. Yet, the interpretation of exercise capacity is challenged by changes in body composition during growth. Our aim was to create an overview of disease-specific exercise capacity in children with CHD. We performed a multicentre retrospective study of exercise capacity of CHD patients, aged 6–18 years, tested between January 2001 and October 2018. Sex-specific distribution graphs were made using the LMS method and height to relate to body size. We included all CHD with *N* > 50, including severe defects (e.g., univentricular heart, tetralogy of Fallot) and “simple” lesions as ventricular septum defect and atrial septum defect. We included 1383 tests of 1208 individual patients for analysis. The peak oxygen uptake (VO_2_peak, 37.3 ml/min/kg (25th–75th percentile 31.3–43.8)) varied between specific defects; patients with univentricular hearts had lower VO_2peak_ compared with other CHD. All groups had lower VO_2peak_ compared to healthy Dutch children. Males had higher VO_2peak_, W_peak_ and O_2_pulse_peak_ than females. Sex- and disease-specific distribution graphs for VO_2peak_, W_peak_ and O2pulse_peak_ showed increase in variation with increase in height.

*Conclusion*: Disease-specific distribution graphs for exercise capacity in children with CHD from a large multicentre cohort demonstrated varying degrees of reduced VO_2peak_ and W_peak_. The distribution graphs can be used in the structured follow-up of patients with CHD to predict outcome and identify patients at risk.

**Table:** 

**What is Known:** • *Children with congenital heart disease (COnHD) are at risk to develop heart failure, arrhytmia’s and other complications. Exercise capacity may be an important predictor for outcome in children with ConHD. In children, the interpretation of exercise capacity poses an additional challenge related to physical changes during growth.*	
**What is New:** • *In this report of a multi-center cohort >1300 childrewn with ConHD, we related the changes in exercise capacity to length. We demonstrated that exercise capacity was reduced as compared with healthy children and we observed variation between disease groups. Patients with a univentricular circulation (Fontan) had worse exercise capacity. We constructed disease specific charts of development of exercise capacity throughout childhood, accessible via a web-site. These graphs may help practitioner to guide children with ConHD.*

**Supplementary Information:**

The online version contains supplementary material available at 10.1007/s00431-022-04648-9.

## Introduction

The last decades have seen major improvements in treatment of patients with congenital heart disease (CHD). The concomitant increase in prevalence of patients surviving with a CHD leads to a new population at risk for major long-term adverse events [1]. Many children treated for CHD have some degree of structural abnormality remaining after surgical correction, which can predispose to develop complications such as arrhythmias or heart failure [2, 3]. These complications may lead to impaired exercise tolerance, re-intervention, rehospitalisation, or death [4, 5].

A major quest in the follow-up of children with CHD is to predict who will develop these complications and when to intervene. In adults, exercise capacity is a good predictor of mortality in Fontan patients [6] and can be used as a surrogate of morbidity [7]. Exercise capacity could also be used in the paediatric population with CHD as it can be serially assessed with a cardiopulmonary exercise test (CPET) [8]. There is a wide variety in types of CHD with potentially different effects on baseline exercise capacity, e.g., univentricular heart, tetralogy of Fallot, transposition of the great arteries, or ventricular septal defect (VSD). The use of exercise capacity in the growing cohort of patients with CHD has been restricted by limited numbers in this wide variety of specific lesions. Recent guidelines state that exercise capacity should be related to disease specific reference values, yet to the best of our knowledge, there is only limited data available for peak oxygen consumption for children with CHD [9, 10].

In children, the interpretation of CPET poses an additional challenge related to physical changes during growth [11]. In general, exercise capacity increases with growth to reach a maximum in early adulthood [12–15]. To avert confounding, there is a need to relate CPET parameters with metrics of growth. An ideal metric is easy to attain and allows comparison of data throughout growth.

Our aim was to provide an overview for exercise capacity for children with CHD. For that purpose, we first tested available metrics related to growth (age, length, weight and BSA) and next analysed CPETs obtained during routine follow-up visits at the outpatient clinic.

## Materials and methods

This study is a retrospective multicentre cohort study of children treated for CHD in four university hospitals, (i) Erasmus Medical Centre — Sophia Children’s Hospital, (ii) University Medical Centre Utrecht — Wilhelmina Children’s Hospital, (iii) Leiden University Medical Centre — Willem-Alexander Children’s Hospital and (iv) Radboud University Medical Centre — Amalia Children’s Hospital. Institutional review boards from all centres approved retrospective data collection.

### Study population

We included children who had performed a CPET between January 2001 and October 2018. These CPETs were conducted in either regular clinical follow-up or in studies performed in one of the centres. We included all patients between the ages of 6 and 18 with a structural heart defect who were able to perform a CPET. We excluded children who underwent and intervention after the CPET (defined as admission to the hospital within 3 months after the exercise test) as these children may represent a group with clinical deterioration. We excluded children with a respiratory exchange ratio (RER) at peak exercise of less than 1.00 as an index of maximal effort. Patients could be included more than once if multiple CPETs were available.

Clinical information on patients was gathered either from the national database KinCor [16] or from the patient information systems from the local hospitals. The CHD diagnosis was classified using the classification system of KinCor, which is based on the ICD classification. Patients with multiple defects were categorized in the most “severe” group; the hierarchy of severity was based on the ICD classification. A detailed description of disease classification is given in the supplement.

We choose a minimum group size of 50 to analyse a group separately. If a group was < *n* = 50, it was combined with a comparable group if possible. Some diagnostic groups were deemed too small to provide reliable overview values, i.e. congenital corrected transposition of the great arteries (*n* = 6), atrioventricular septal defect (*n* = 30), pulmonary atresia with VSD classified in the group of tetralogy of Fallot (*n* = 12), tricuspid valve abnormalities (*n* = 49) and mitral valve abnormalities (*n* = 29). The latter two were combined to atrioventricular (AV) valve abnormality; the others were excluded. All children with transposition of the great arteries were operated using an arterial switch operation, and all children with an ASD and VSD were operated.

### Exercise tests

All exercise tests were performed on a cycle ergometer in the upright position, while breath-by-breath gas analyses were done. Cycle ergometry is the most used form of exercise testing in Europe. In all centres, the Godfrey protocol was used, which consists of a 3-min warm-up period followed by a progressive increase in workload depending on the height of the child: 10 W/min for children less than 120 cm in height, 15 W/min for children from 120 to 150 cm and 20 W/min for children over 150 cm until failure. During this increase in workload, continuous electrocardiography and ventilation measurements are taken [17]. Exact test equipment is given in the online supplement. All tests were overseen by a medical qualified person to conduct these tests.

VO_2peak_ was defined as the highest VO_2_ measured over an average of 30 s. W_peak_ was defined as the highest average value for the last 30 s. Peak heart rate was defined as the highest heart rate achieved averaged over a total of 10 s, and O_2_pulse was defined as VO_2peak_/heart rate for the last 30 s. For predicted VO_2peak_,W_peak_ and peak heart rate (HR_peak_) data from 214 healthy Dutch children between 8 and 18 years old were used [12, 18]. VE/VCO_2_ slope was calculated until peak exercise.

### Statistics

#### Quality control and standardization methods

We analysed the best metric to standardize the reference values. We included the following metrics: age, height, weight and body surface area (BSA) in our analyses and stratified the analyses for sex. We calculated BSA using the Haycock formula. We tested differences between models using VO_2peak_ (ml/min) and peak workload (W_peak_) as outcome parameters. Differences between models were tested using a log likelihood test.

#### Baseline characteristics and LMS curves

We tested all variables for normal distribution visually with histograms, q-q plots and using the Shapiro-Wilcox test. Variables which are normally distributed are shown as mean ± standard deviation (SD) and parameters which are not normally distributed are displayed as median and 25th–75th percentile. We tested all CPET outcome parameters with height using Spearman’s correlation. Those with a significant correlation are shown as graphs, and those without are shown as mean ± standard deviation or median and 25th–75th percentile. Differences between groups are tested with ANOVA and post hoc analysis with the Tukey test. To construct the reference value graphs, we used the lambda mu sigma (LMS) method described by Cole et al. [19]. All analyses (except for constructing the LMS charts) were done in R version 3.4.4, and LMS Chartmaker Light by Cole et al. was used to construct LMS models [20].

## Results

### Patient characteristics

We included 1383 tests of 1208 individual children for analysis (Suppl. Figure 1) with ASD, VSD, atrioventricular (AV) valve abnormality, pulmonary stenosis, aortic stenosis, aortic coarctation, transposition of the great arteries, tetralogy of Fallot and univentricular hearts. Patients could be included more than once in this cohort. Patient characteristics are shown in the supplement (Suppl. Table 1). Of the patients, 57.5% were male, the median age was 13.3 years (25–75% range 7.2–18.0), the median weight was 48.0 kg (25–75% range 37.3–58.6), and the median height was 160.3 cm (25–75% range 149.0–170.0).

### Standardization method

To obtain an easy and reliable metric, we assessed the *R*^2^ for the parameters VO_2peak_ and W_peak_ using the metrics: age, height, weight and BSA, stratified for sex (Suppl. Figure 1). Of these metrics, height, weight and BSA performed significantly better than age. This pattern was similar for both sexes (male and female) and both outcome parameters (VO_2peak_ and W_peak_). There were small but statistically significant differences: height performed better in explaining variance in W_peak_ whereas weight and BSA performed slightly better in explaining variance in VO_2peak_. We decided to use height from here on because BSA is more complicated to compose, and many different formulas are used [21]. Also, height is known to be more consistent in the population over time. For example, the height of Dutch children has not changed over the last 20 years whereas there has been a substantial rise in obesity rates complicating the use of weight [22].

### Exercise parameters

There was a large and significant variation in exercise capacity between disease groups (Tables 1 and 2). Patients with a univentricular heart performed worse than all other patient groups, except for AV valve abnormalities in VO_2peak_ and W_peak_ (Suppl. Table 2–10 shows ANOVA with corresponding Tukey tests). VE/VCO_2_ was elevated in patients with univentricular hearts as compared with all other diagnostic groups (Table 1; Fig. 2, supplementary Fig. 3–8).

**Table 1:** Exercise parameters 1/2

		**VO**_**2peak**_		**VO**_**2peak**_**/kg**				**W**_**peak**_		**W**_**peak**_**/kg**			**HR**_**peak**_			**VE/CO**_**2**_	
	*N*	Median (ml/min)	(25th–75th percentile)	Median (ml/min/kg)	(25th–75th percentile)		Median (W)		(25th–75th percentile)	Median (W/kg)	(25th–75th percentile)	Median (beats/min)			(25th–75th percentile)	Median	(25th–75th percentile)
**ASD**	70	1819	(1496–2173)	37.4	(31.4–43.8)		158		(120–180)	3.0	(2.6–3.4)	187			(178–195)	29.4	(26.0–33.0)
**VSD**	180	1674	(1324–2084)	37.7	(32.6–42.3)		142		(105–179)	3.1	(2.6–3.5)	182			(171–190)	27.0	(24.0–31.2)
**AV valve abnormality**	78	1512	(1213–1878)	34.1	(27.3–42.4)		133		(100–160)	3.0	(2.3–3.3)	182			(172–193)	30.3	(27.0–42.5)
**Aortic stenosis**	187	1908	(1574–2381)	40.6	(34.7–46.9)		156		(120–200)	3.2	(2.8–3.8)	185			(178–192)	28.5	(23.7–31.3)
**Pulmonary stenosis**	80	1841	(1444–2471)	39.4	(33.4–44.1)		144		(109 199)	3.0	(2.6–3.5)	184			(173–190)	30.4	(25.9–35.2)
**Aortic coarctation**	141	1782	(1462–2264)	39.6	(33.8–45.3)		140		(120–184)	3.1	(2.7 -3.6)	184			(173–190)	29.5	(23.1–35.2)
**Transposition of the great arteries**	182	1805	(1438–2251)	39.6	(32.0–45.2)		160		(120–198)	3.2	(2.7–3.6)	182			(173–187)	29.5	(25.9–33.5)
**Tetralogy of Fallot**^**a**^	252	1669	(1325–2100)	36.4	(31.3 -43.2)		135		(103–180)	3.0	(2.5–3.4)	180			(171–187)	26.7	(23.0–32.5)
**Univentricular heart**	213	1354	(1144–1596)	32.1	(25.8 -37.7)		105		(90–136)	2.5	(2.0 -2.9)	174			(160–184)	35.5	(31.3–42.4)
**Total**	1383	1669	(1341–2133)	37.3	(31.3–43.8)		140		(105–180)	3.0	(2.5–3.5)	181			(171 -190)	29.8	(25.0–35.0)

Values are medians (25th–75th percentile)

*ASD* atrial septal defect, *VSD* ventricular septal defect, *AV* atrioventricular

^a^Tetralogy of Fallot or pulmonary atresia with VSD

**Table 2:** Exercise parameters 2/2

	**VO**_**2peak **_**as % of predicted**		**W**_**peak**_** as % of predicted**		**HR**_**peak**_** as % of predicted**		**O**_**2**_** pulse**_**peak**_		**RER max**	
	Median	(25th–75th percentile)	Median	(25th–75th percentile)	Median	(25th–75th percentile)	Median	(25th–75th percentile)	Median	(25th–75th percentile)
**ASD**	84	(71–93)	88	(75–98)	99	(94 -103)	10.3	(8.0–11.6)	1.15	(1.09–1.21)
**VSD**	77	(64– 93)	82	(67–96)	96	(90–101)	9.4	(7.6–11.3)	1.14	(1.09–1.20)
**AV valve abnormality**	70	(54–82)	70	(60–84)	97	(92–102)	8.5	(6.5–10.3)	1.14	(1.08–1.22)
**Aortic stenosis**	86	(70–99)	85	(73–99)	97	(94–101)	10.5	(8.4–12.8)	1.15	(1.10–1.21)
**Pulmonary stenosis**	81	(70–91)	79	(68–90)	97	(91–100)	10.4	(7.7–13.2)	1.13	(1.08–1.20)
**Aortic Coarctation**	85	(72–97)	84	(70–96)	97	(91–101)	9.8	(8.1–12.5)	1.15	(1.09–1.21)
**Transposition of the great arteries**	79	(66–97)	85	(69–97)	96	(91–99)	10.0	(8.3–12.5)	1.15	(1.09–1.22)
**Tetralogy of Fallot**	75	(62–88)	77	(66–91)	95	(90–99)	9.4	(7.4–11.9)	1.14	(1.09–1.21)
**Univentricular heart**	63	(53–73)	63	(53–72)	92	(85–97)	7.8	(6.7–9.3)	1.11	(1.06–1.15)
**Total**	76	(62–91)	78	(65–93)	96	(90–100)	9.4	(7.5–11.8)	1.14	(1.09–1.20)

Values are medians (25th–75th percentile)

*ASD* atrial septal defect, *VSD* ventricular septal defect, *AV* atrioventricular

**Fig. 1:**
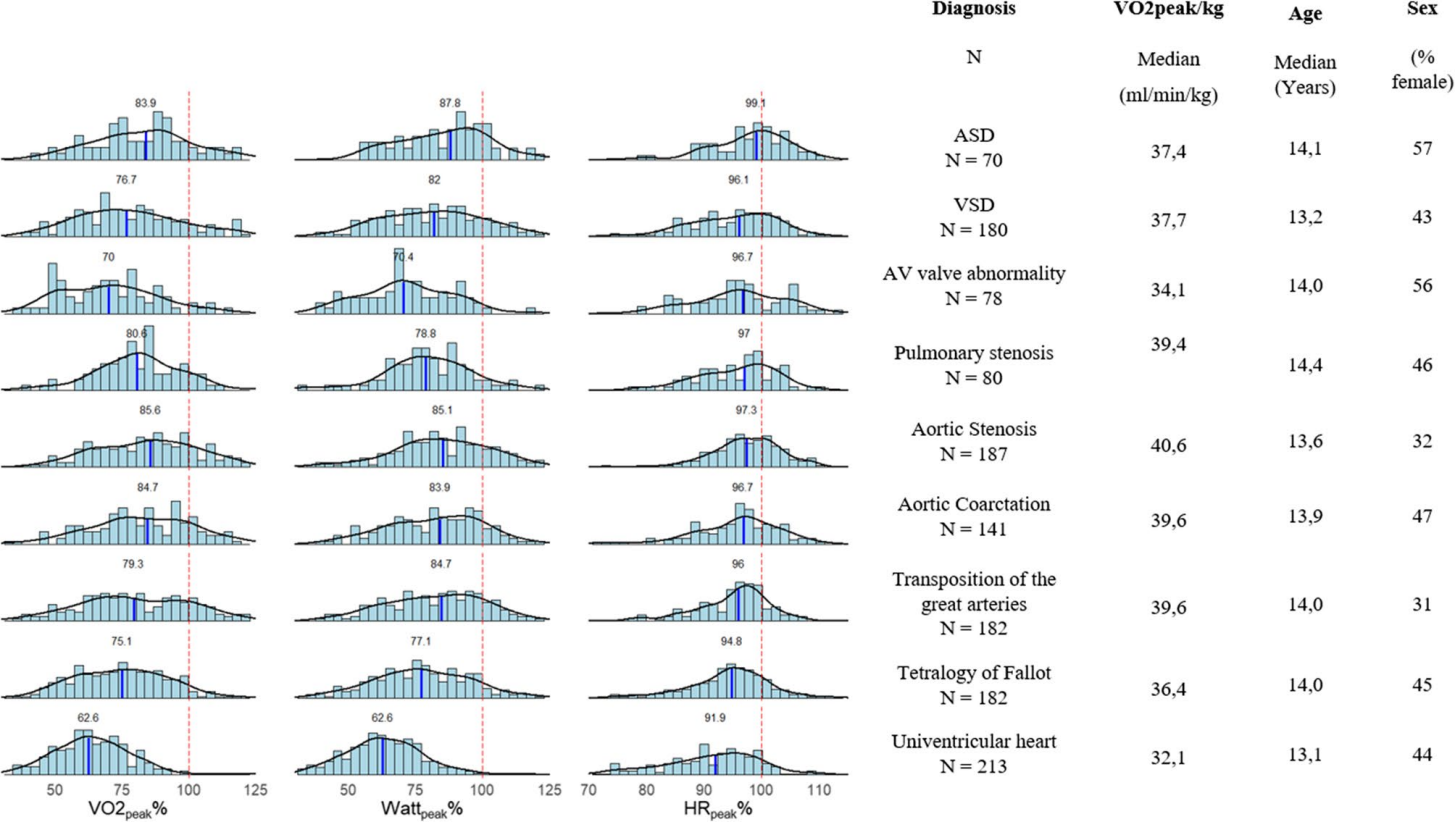
Histograms of PeakVO_2_, peak workload and peak heart rate as percentage of predicted in the different disease groups. The number above the histogram represents the median. The red dotted line represents 100%. On the right side the number of patients per group, median VO2/kg, median age of the group and distribution of sex is given

All patient groups had lower VO_2peak_, W_peak_ and HR_peak_ as percentage of predicted based upon an age-matched healthy control group; again, patients with a univentricular heart performed worse compared to all other patient groups except for AV valve abnormalities (Fig. 1) [12]. VO_2peak_, as predicted for their age, ranged from 86% (25–75% range 70%–99%) in patients with aortic stenosis to 63% (25–75% range 53–73%) in patients with univentricular hearts. W_peak_ as percentage of predicted ranged between 88 (25–75% range 75–98%) for patients with ASDs and 63% (25–75% range 53–72%) in patients with univentricular hearts. The lowest median HR_peak_ was 92% (25–75% range 85–97%) of predicted in patients with univentricular hearts (*p* < 0.001). All percentages of predicted are displayed graphically in Fig. 1.

To relate our results to body size, we constructed disease-specific graphs and values using height as metric.

**Fig. 2:**
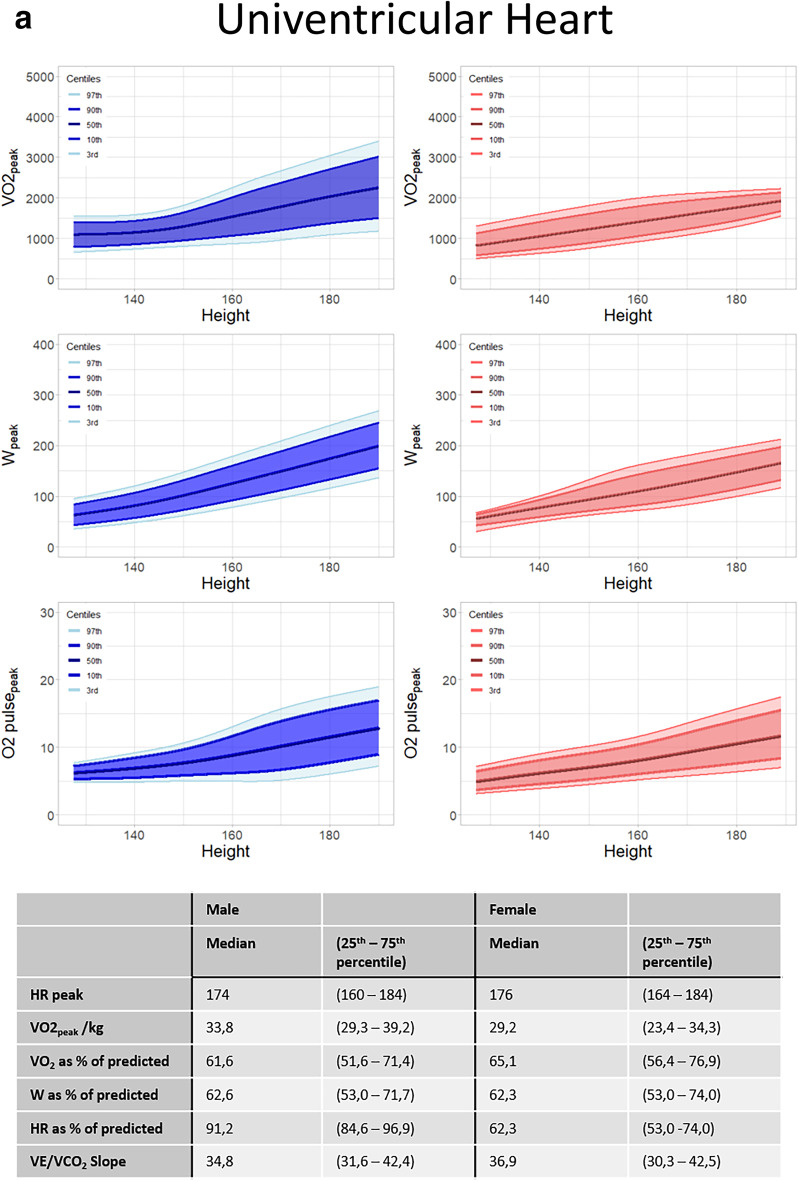

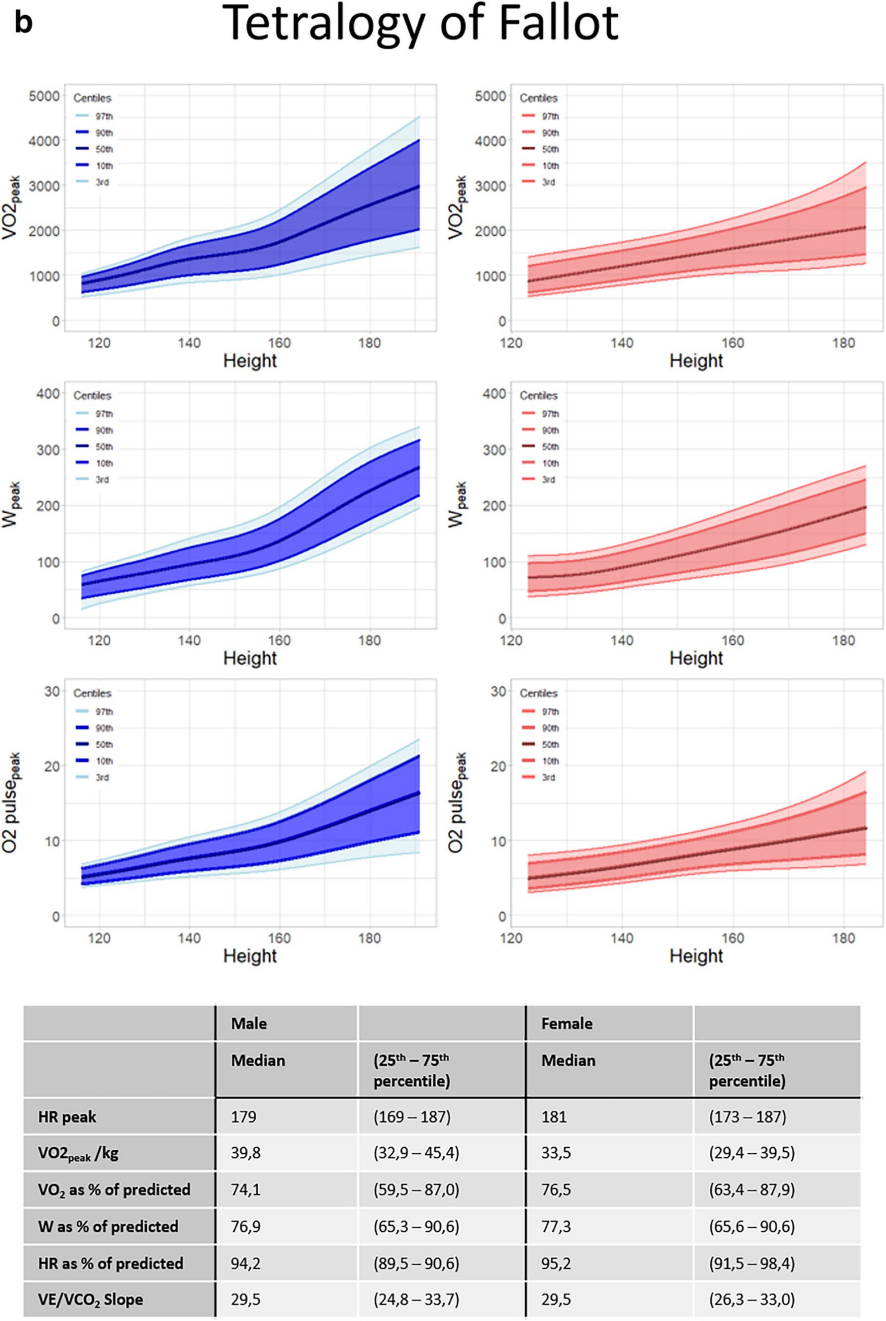

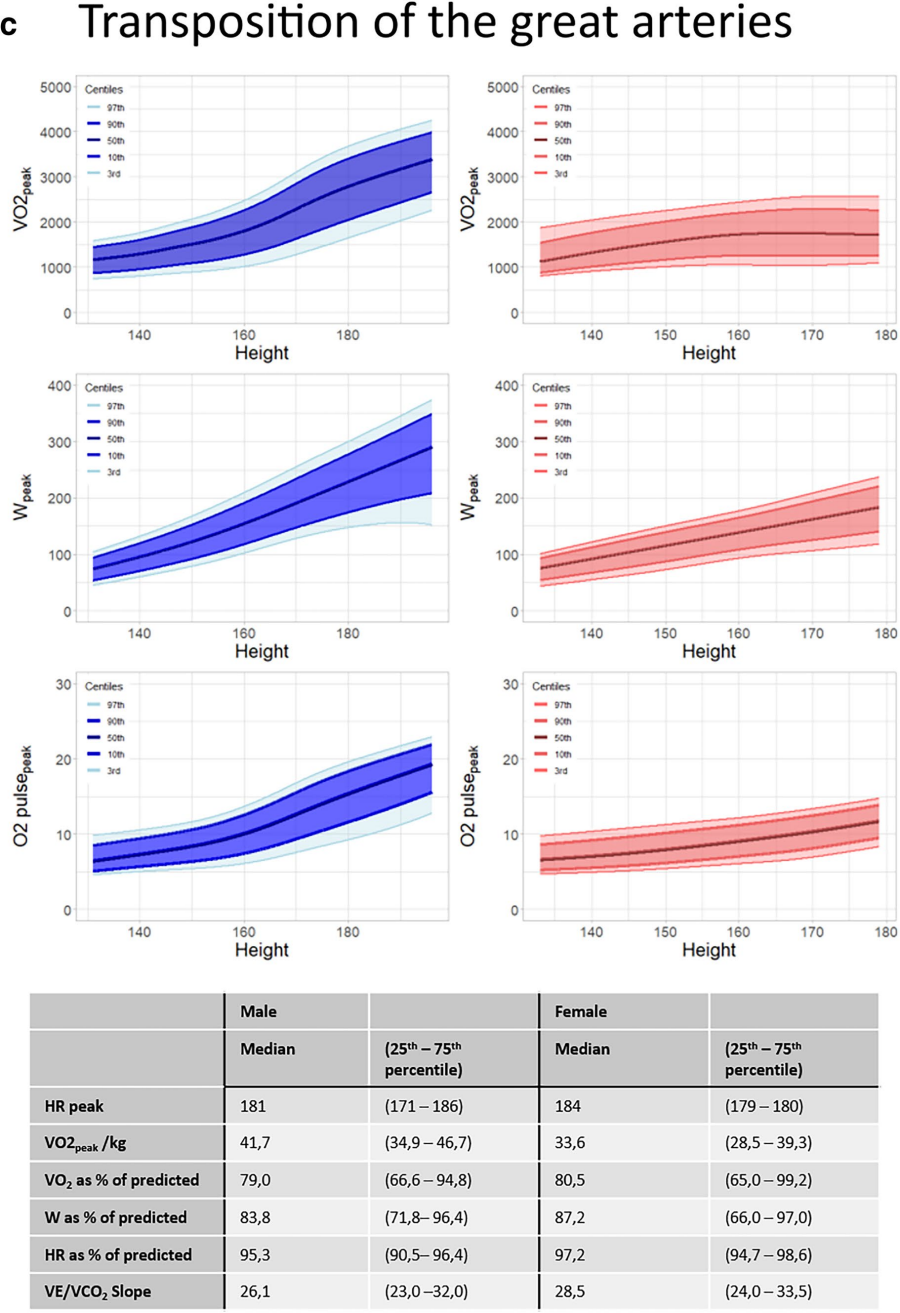
**a** Sex-specific distribution graphs for VO_2peak_, W_peak_ and O2pulse_peak_ and median HR_peak_, VO_2peak_/kg, VO_2_ as% of predicted, W_peak_ as % of predicted, HR as % of predicted and VE/VCO2 slope in patients with Univentricular hearts related to height (in cm). Dark blue/dark red areas represents 80 centiles, lighter shade represents 95 centiles. Table at the bottom shows median and 25-75 quantiles. **b** Sex-specific distribution graphs for VO_2peak_, W_peak_ and O2pulse_peak_ and median HR_peak_, VO_2peak_/kg, VO_2_ as% of predicted, W_peak_ as % of predicted, HR as % of predicted and VE/VCO2 slope in patients with tetralogy of Fallot related to height (in cm). Dark blue/dark red areas represents 80 centiles, lighter shade represents 95 centiles. Table at the bottom shows median and 25-75 quantiles. **c** Sex-specific distribution graphs for VO_2peak_, W_peak_ and O2pulse_peak_ and median HR_peak_, VO_2peak_/kg, VO_2_ as% of predicted, W_peak_ as % of predicted, HR as % of predicted and VE/VCO2 slope in patients with transposition of the great arteries related to height (in cm). Dark blue/dark red areas represents 80 centiles, lighter shade represents 95 centiles. Table at the bottom shows median and 25-75 quantiles

Figure 2 shows the distribution graphs for VO_2peak_, W_peak_ and O2pulse_peak_ for males and females separately in patients with univentricular hearts (Fig. 2a), tetralogy of Fallot (Fig. 2b) and transposition of the great arteries (Fig. 2c). All distribution graphs can be found and downloaded at “https://pedcardio.shinyapps.io/Racer2/”. Reference plots for all disease groups are in the supplement (Suppl. Figure 3–8) The parameters HR_peak_, VO_2peak_/kg, VO_2peak_ as % of predicted, W_peak_ as % of predicted, HR_peak_ as % of predicted and the last 30 s 2 were not significantly associated with height and therefore shown as a median with 25th and 75th percentile in the table as follows. For all parameters, males had higher values compared with females. Also, the variance of the data (displayed by the centile curves) increased with height.

In patients with univentricular hearts, the VO_2peak_/kg decreased with age (0.97 ± 0.23 ml/kg/min per year), and in patients with biventricular circulations, there was no association between age and VO_2peak_/kg.

Lastly, we analysed the effect of left ventricular dominance vs right ventricular dominance in patients with univentricular circulations. Patients with right ventricular dominance had lower VO_2peak_ as % of predicted (67.8 [57.9–75.1] vs. 60.3 [52.6–68.3], *p* = 0.015) and W_peak_ as % of predicted (65.5 [57.2–74.2] vs. 62.1 [52.2–68.4], *p* = 0.025). No differences in HR as % of predicted were observed.

## Discussion

In this study, we present the exercise capacity in a large cohort of children with CHD. In healthy children, exercise capacity increases with the increase in body size during childhood. To construct graphs of the observed distribution in exercise capacity in children with CHD, we related the exercise parameters to height. Exercise capacity was reduced in all CHD groups as compared to the normal population, and we observed a large variation between different groups of CHD. Therefore, we constructed disease-specific distribution graphs. These graphs and values can be used to guide clinicians during the serial follow-up of patients with CHD, e.g. in transition to adult services, and can be used as an indication for abnormal changes in CPET results and emphasize the need for identification of the cause, such as cardiac or other disease or inadequate changes in physical activity. There is a need for large datasets of exercise capacity to identify patients at risk for adverse outcome. Recent studies suggest that lack of normal development of exercise capacity during children can predict worse outcome [23]. As we have come increasingly aware of the potential of preventive strategies to postpone long-term complications in CHD, it is important to analyse exercise capacity of patients with CHD during childhood growth. Previous studies of exercise capacity in adults with CHD, covering a large age range of patients treated in a different era, although valuable, did not account for the change in body size and growth during childhood [24].

In comparison with previously published data, we observed similar VO_2peak_/kg and lower VO_2peak_ as percentage of predicted in most patient groups [10, 25–28]. These differences may be due to the use of a different reference group. Several studies [10, 25, 27] used the data published in 1984 by Wasserman and Cooper, in which 107 American children were studied [15]. We used a recent Dutch cohort of 214 children described in 2011 [12, 13]. It should be noted that in patients with a univentricular circulation, in general, European cohorts describe a higher VO2/kg than cohorts from USA and Japan (~ 32 ml/kg/min vs. ~ 27 ml/kg/min) [25–27]. In the cohort described by Paridon et al., only 166 out of 411 patients had been able to complete a maximal exercise test. There are large differences in cohort size, ranging from 25 to 311 patients with a univentricular circulation. These differences may also explain different results when comparing patients with left ventricular dominance with those with right ventricular dominance. In this cohort, exercise capacity was lower in patients with right morphology dominant univentricular hearts. Smaller size cohorts of univentricular hearts have found different results, but on average, most large size cohorts showed similar differences [29].

In adults with a univentricular circulation, a decrease in VO_2peak_ as percentage of predicted of > 3% per year has been shown to be a predictor for death or cardiac surgery [30]. In the present study, we describe a similar decrease in VO_2_ peak/kg in patients with a univentricular circulation, and recently, Janousek et al. described a similar decrease in a group of patients ranging from 10 to 30 years of age [31]. Follow-up studies in this cohort are necessary to determine the effects of attrition on outcome. It should be noted that VO_2_ peak (in ml/min) increases with height during childhood in the normal population but also in children with CHD. The use of VO_2peak_ in ml/min plotted versus height until adulthood and versus age thereafter would facilitate comparison as well as transition into adult care services. Thus, the use of disease specific and growth specific exercise capacity graphs can identify deviations from expected development of VO_2peak_. Predictive value of exercise capacity for cardiac-related hospitalization has been demonstrated in one study of children with tetralogy of Fallot [8] and in several studies in patients > 18 year of age. Further research is needed to identify personalized risk scores for adverse outcome using development of exercise capacity in addition to other clinical parameters.

In this study, all patients with CHD had a decreased exercise capacity, even patients with lesions that are deemed less severe such as an ASD and VSD. Previous studies in adults also indicated that despite excellent survival, exercise capacity in “simple lesions” was not normal [24, 32]. There is no obvious explanation for these findings, e.g. there was no limitation to increase peak heart rate. For patients with a VSD, a dysfunctional septal architecture has been suggested to be involved [33]. For patients with an ASD, timing of closure and hence duration of increased RV preload may affect the ability for remodelling [34]. The results of several studies to exercise capacity in”simple” CHD lesions favour a more rigorous follow up of “simple lesions” during childhood and beyond.

There is a discussion in paediatrics which metric to use to standardize exercise capacity during growth. A systematic review in 2015, describing six different metrics to standardize exercise capacity in paediatrics (age, height, weight, BSA, lean body mass and pubertal stage), did not provide a conclusion which metric best corrects for differences in body size [35]. Most studies used age to standardize CPET in childhood, as in adults [35, 36]. Yet, in adults, VO_2peak_ and W_peak_ decrease with age, whereas body composition is relatively stable [24, 37]. In contrast, in children, VO_2peak_ and W_peak_ increase with age and the main factors seem to be the development and growth of muscle mass, lung capacity, and cardiac output [12]. The findings of our study would suggest the use of height, weight or BSA rather than age to standardize exercise capacity in children with CHD. Our observations are in line with a previous study assessing reference values for the 6-min walk test [38]. In comparison with body weight, height is relatively unaffected by obesity. Thus, to reduce variation, we choose height over weight and BSA as metric to standardize CPET.

The strength of our study is the number of children with CHD included. This allowed us to construct disease-specific distribution graphs. Another strength is that we included all CPETs conducted and therefore have a good representation for day-to-day clinical CPETs. Yet, this study design also comes with some limitations; particularly, the inclusion of all CPETs may also lead to selection bias. Our cohort included a range of different CHD, with an overrepresentation of the more severe conditions such as tetralogy of Fallot and univentricular hearts [39]. In these patients in general, more rigorous follow-up surveillance is performed. To achieve adequately sized groups, we had to combine heterogenic groups such as mitral and tricuspid valve abnormalities, although these conditions may have very different physiologies. Furthermore, we were not able to add clinical data such as echocardiograms and MRIs to our study. Also, we did not select or correct for medication use of our patients, specifically beta blockers which may limit peak exercise performance [40]. In adult cohorts, the percentage of patients using beta blockers is ~ 15% [41]. In our study, peak heart rate was > 95% in most groups, with exception for the patients with univentricular hearts. In patients with univentricular heart, a reduced peak heart rate has been described previously and is not related to beta blocker use, rather to abnormal physiology [42]. We justify this approach, since excluding these patients may have yielded another selection bias. Also, we did not correct for any other diseases, socioeconomic or life-style factors including sport habits. Our data are largely in agreement with data from the literature; hence, these effects probably are either small or bias to every study. The positive result of this approach is that our cohort is representative of a present-day cohort of children of CHD. Lastly, the lack of ethnic diversity in our cohort may reduce the generalizability. Our data applies to children from Caucasian background predominantly. In order to circumvent this problem of variance between ethnic groups, we used height to relate CPET outcomes to body size. This is important since the Dutch are amongst the tallest of human populations in the world. Expressing the CPET results per height makes our value set usable in other Caucasian population outside of the Netherlands.

In conclusion, we constructed disease-specific distribution graphs for exercise capacity in children with CHD during growth and development using a relatively large national multi-centre cohort. Children with CHD had reduced exercise capacity varying with specific disease, thereby justifying disease-specific reference values. These values can be used in the structured follow-up of children with CHD, in transition to adult services, and can be used to better identify lack of physical activity.

## Supplementary Information

Below is the link to the electronic supplementary material.

Supplementary file1 (PDF 2261 KB)

## Abbreviations

ASD: Atrial septum defect
BSA: Body surface area
CHD: Congenital heart diseases
CPET: Cardio pulmonary exercise test
RER: Respiratory exchange ratio
SD: Standard deviation
VSD: Ventricular septum defect
