# Supplementary material for: Exercise capacity in a cohort of children with congenital heart disease
Source: Eur J Pediatr. 2022 Nov 5;182(1):295–306. doi: 10.1007/s00431-022-04648-9 (PMC9829639; doi:10.1007/s00431-022-04648-9)
Supplement: Supplementary file 1 — Supplementary file1 (PDF 2261 KB) [file 431_2022_4648_MOESM1_ESM.pdf]

Online supplement

Reference values for exercise capacity in children with congenital heart disease.

Wouter J. van Genuchten MD, MSc [a, e]; Willem A. Helbing MD, PhD [a, c]; Arend.D.J. ten Harkel MD, PhD [d]; Zina Fejzic, MD [c]; Irene M. Kuipers MD, PhD [e]; Martijn G. Slieker; MD, PhD [h]; Jelle P.G. van der Ven MD [a, f]; Eric Boersma MSc, PhD [b]; Tim Takken, MSc, PhD [g]; Beatrijs Bartelds MD, PhD [a]

Department of Pediatric Cardiology [a], Department of Cardiology [b] Erasmus MC, University Medical Center, Rotterdam, the Netherlands; Department of Pediatric Cardiology [c], Radboud University Medical Center, Nijmegen, the Netherlands; Department of Pediatric Cardiology [d], Leiden University Medical Center, Leiden, the Netherlands; Department of Pediatric Cardiology [e], Amsterdam University Medical Center, Amsterdam, the Netherlands; Netherlands Heart Institute [f]; Department of Medical Physiology [g], Department of Pediatric Cardiology [h], Wilhelmina Children's Hospital, University Medical Center Utrecht, Utrecht, the Netherlands

Table of content

Supplement 1: CPET protocol per center

Supplement 2: Quality control

Supplemental Figure 1: R-squared values for W peak and VO_2 peak

Supplemental Figure 2: selection of study population

Supplemental Figure 3: reference values in patients with Aortic stenosis, in males and females separately.

Supplemental Figure 4: reference values in patients with transposition of the great arteries, in males and females separately.

Supplemental Figure 5: reference values in patients with ASD, in males and females separately.

Supplemental Figure 6: reference values in patients with AV valve abnormality, in males and females separately.

Supplemental Figure 7: reference values in patients with Pulmonary stenosis, in males and females separately.

Supplemental Figure 8: reference values in with Aortic Coarctation, in males and females separately.

Supplemental table 2: Tukey analysis of VO_{2peak}

Supplemental table 3: Tukey analysis of VO_{2peak}/kg

Supplemental table 4: Tukey analysis of W_{peak}

Supplemental *table 5:* *Tukey analysis* of W_{peak} /kg

Supplemental table 6: Tukey analysis of HR_{peak}

Supplemental table 7: Tukey analysis of VO_{2peak} as % of predicted

Supplemental table 8: Tukey analysis of W_{peak} as % of predicted

Supplemental table 9: Tukey analysis of HR_{peak} as % of predicted

Supplemental table 10: Tukey analysis of O_2 $pulse_{peak}$

Supplemental Figure 9: Coding system KinCor.

CPET protocol per center

All exercise tests were performed on a cycle ergometer in the upright position: Jaeger ER9000 (Viasys Healthcare, Hoechberg, Germany) in Rotterdam and Leiden, Lode Corival (Lode BV, Groningen, the Netherlands) in Utrecht, Ergoselect 200P (Vyaire Medical, Hoechberg, Germany) in Nijmegen. Breath-by-breath gas analyses was done using an Oxycon Champion System (Viasys Healthcare, Conshohocken, United States) in Rotterdam, ZAN 600 (Accuramed BVBA, Lummen, Belgium) in Utrecht, Masterscreen CPX (Vyaire Medical, Hoechberg, Germany) in Nijmegen and Leiden. Heart rate was measured by a continuous 12 lead ECG. In all centres the test started with 3 minutes of unloaded cycling after which the workload was increased based on their height by 15 or 20 watt per minute (smaller or larger than 150 cm respectively). In Rotterdam a stepwise increase was used (28), in Utrecht both ramp and stepwise increase were used (29), in Nijmegen a ramp protocol was used. Before each exercise test weight and height were measured on calibrated scales and measuring stick. All tests were overseen by a medical qualified person to conduct these tests.

Quality Control

In two centres the full test data was collected and from these values at peak were extracted, in two centres summary reports including peak values were extracted. We routinely checked input from 1/100 files (randomized) and manually inspected all tests with a Z score larger than 2.5 or smaller than -2.5 for the parameters VO_{2peak}, W_{peak} and maximum heart rate (HR_{peak}) for irregularities during the test.

Supplemental table1: patient characteristics

	Count	Sex	Age		Weight		Height		BSA	
	N	Male (%)	Median (years)	(25th-75th percentile)	Median (kg)	(25th-75th percentile)	Median (years)	(25th-75th percentile)	Median (years)	(25th-75th percentile)
ASD	70	43	14.1	(12,0 - 15,5)	51.0	(41.5 - 58.2)	161.5	(151.8 - 170.0)	1.49	(1.31 - 1.67)
VSD	180	57	13.1	(11.7 - 14.8)	46.3	(38.5 - 57.5)	159.0	(149.3 - 168.0)	1.43	(1.25 - 1.62)
AV valve abnormality	78	44	14.0	(12.2 - 15.7)	47.0	(37.3 - 57.7)	157.6	(148.6 - 167.6)	1.42	(1.25 - 1.63)
Pulmonary stenosis	80	54	14.4	(12.1 - 16.3)	50.9	(37.0 - 61.7)	162.0	(149.8 - 174.0)	1.45	(1.23 - 1.72)
Aortic stenosis	187	68	13.6	(11.9 - 15.6)	47.3	(38.0 - 61.6)	162.5	(149.0 - 171.0)	1.53	(1.24 - 1.71)
Aortic Coarctation	141	53	13.9	(11.9 - 15.8)	49.5	(38.1 - 62.0)	161.0	(147.0 - 171.0)	1.49	(1.24 - 1.71)
Transposition of the great arteries	182	69	14.0	(12.2 - 15.6)	50.0	(37.1 - 59.0)	161.8	(152.6 - 171.0)	1.46	(1.30 - 1.67)
Tetralogy of Fallot	252	55	14.0	(11.8 - 15.9)	48.0	(41.0 - 58.2)	164.0	(149.9 - 171.0)	1.50	(1.25 - 1.66)
Univentricular heart	213	56	13.1	(11.4 - 15.6)	43.3	(36.0 - 54.0)	155.0	(147.0 - 166.0)	1.35	(1.20 - 1.56)
Total	1,383	58	13.7	(11.8 - 15.7)	48.0	(37.3 - 58.6)	160.3	(149.0 - 170.0)	1.45	(1.24 - 1.66)

Abbreviations: ASD: Atrial septal defect; VSD: Ventricular septal defect; AV: Atrioventricular; BSA: Body surface area ; Values are medians (25th – 75th percentile) or percentages.

Supplemental figure 1: R-squared for LMS models using different metrics

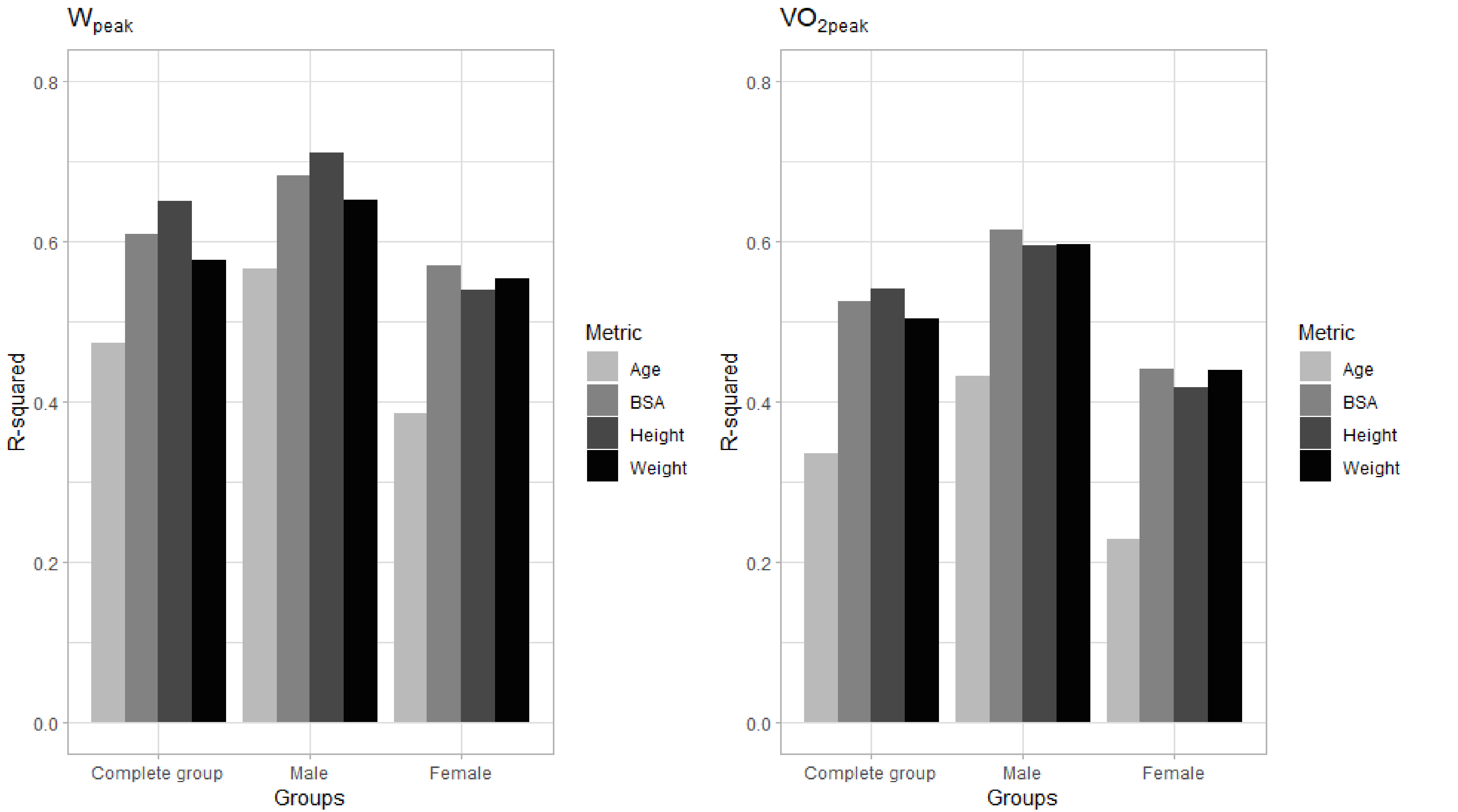

Supplemental figure 2: selection of study population

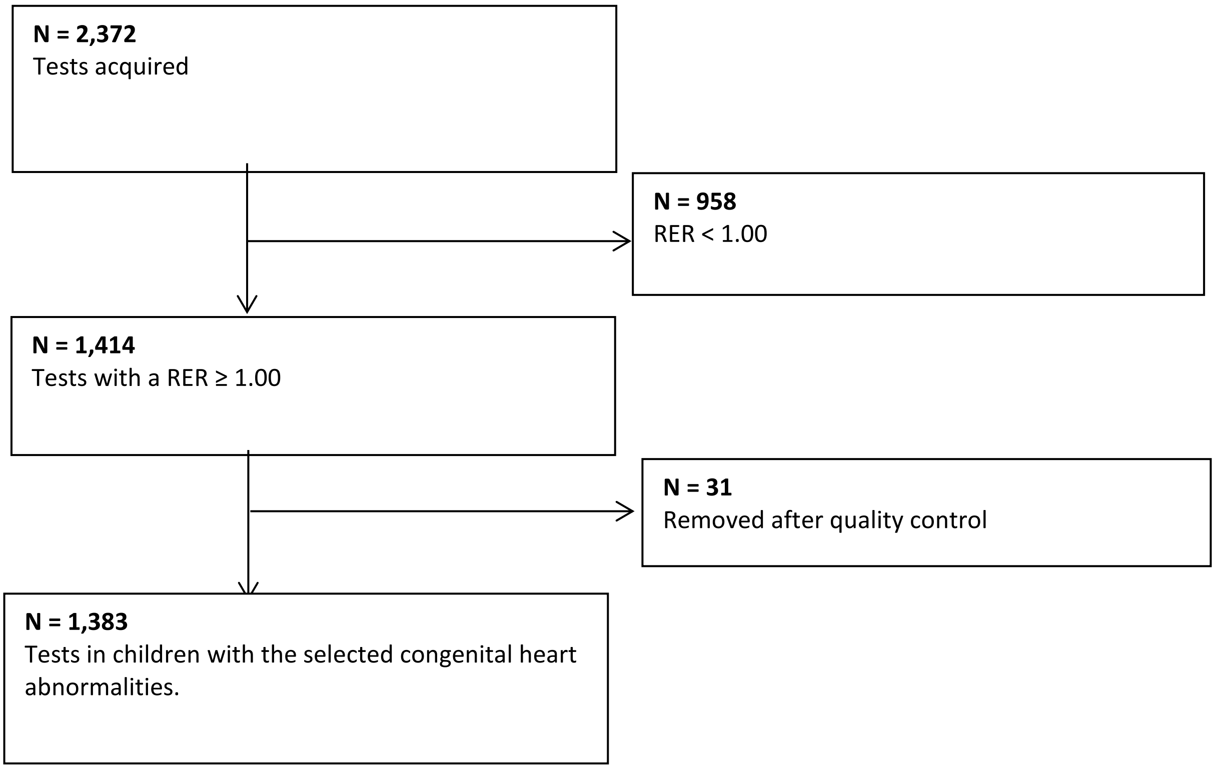

Supplemental figure 3: reference values for $VO2_{peak}$, W_{peak} and $O2pulse_{peak}$ and median HR_{peak}, VO_{2peak}/kg, VO_2 as% of predicted, Wpeak as % of predicted and HR as % of predicted in patients with Aortic stenosis, in males and females separately.

Aortic Stenosis

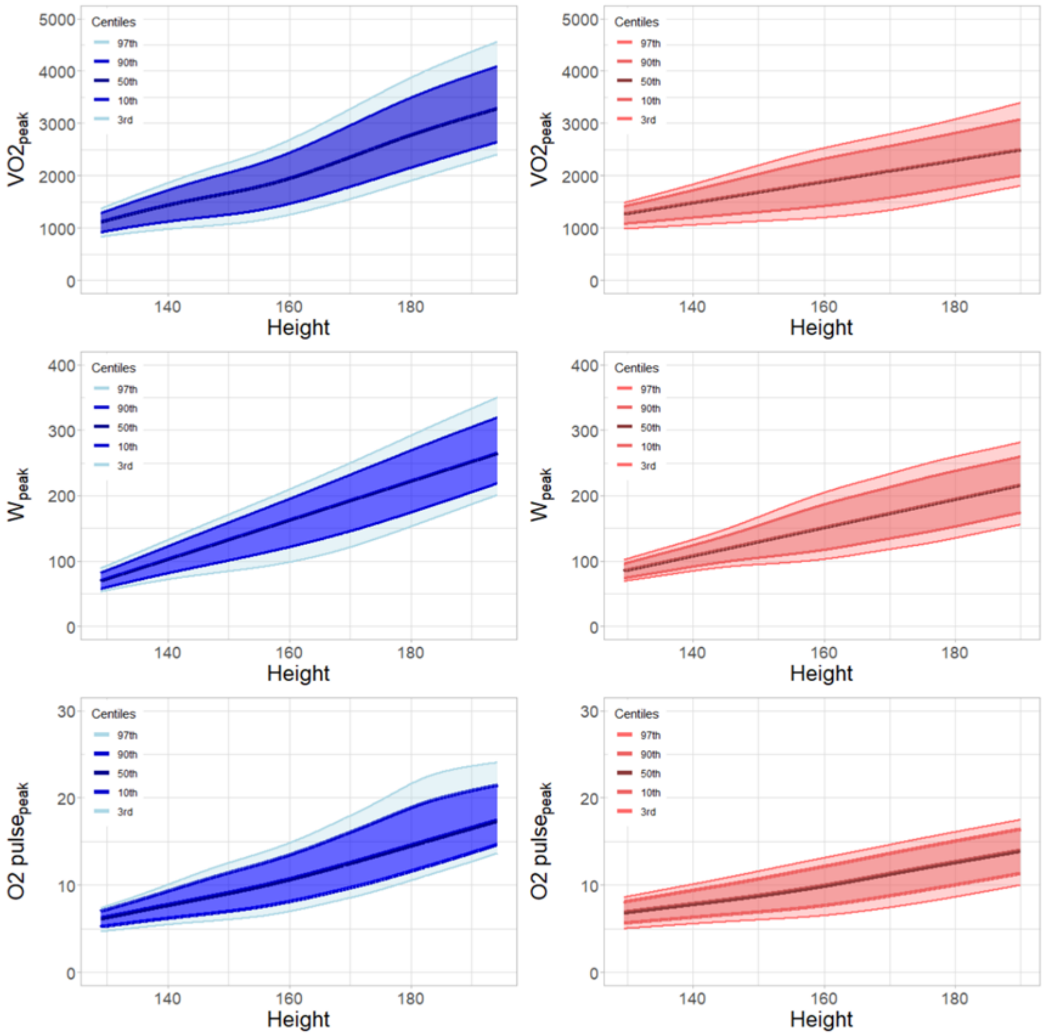

	Male		Female	
	Median	(25th – 75th percentile)	Median	(25th – 75th percentile)
**HR peak**	184	(178 – 190)	188	(179 – 193)
**$VO2_{peak}$ /kg**	44,1	(36,9 – 48,8)	37,4	(31,6 – 41,3)
**VO_2 as % of predicted**	83,6	(67,9 – 95,9)	89,7	(78,7 – 103,5)
**W as % of predicted**	83,2	(71,3 – 97,2)	89,8	(76,5 – 99,1)
**HR as % of predicted**	96,9	(71,3 – 97,2)	89,8	(76,5 – 99,1)
**VE/VCO_2 Slope**	27,2	(23,6 –31,2)	30,0	(25,6 – 32,6)

Supplemental figure 4: reference values for VO_{2peak}, W_{peak} and $O2pulse_{peak}$ and median HR_{peak}, VO_{2peak}/kg, VO_2 as% of predicted, Wpeak as % of predicted and HR as % of predicted in patients with VSD in males and females separately.

VSD

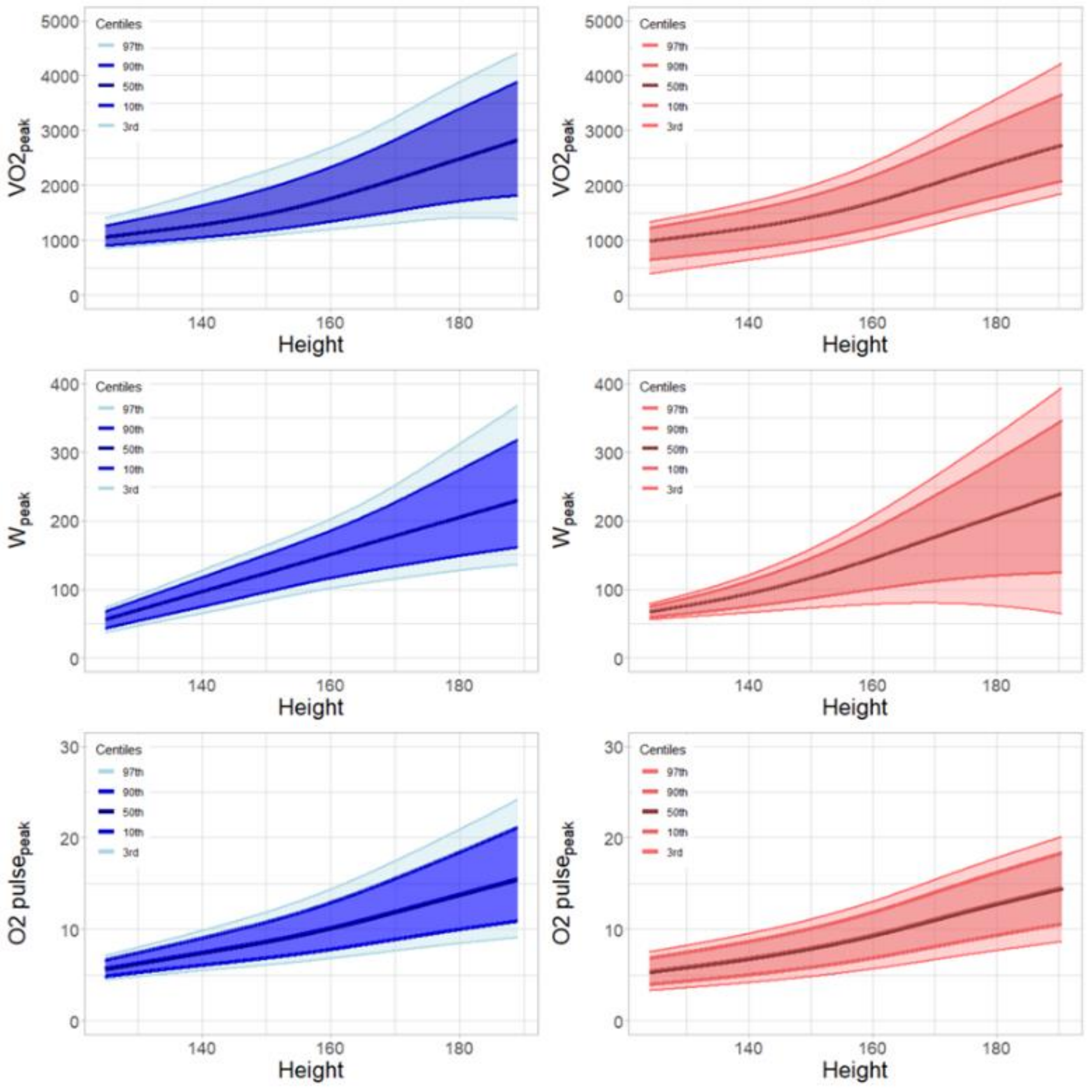

	Male		Female	
	Median	(25th – 75th percentile)	Median	(25th – 75th percentile)
**HR peak**	180	(169 – 190)	184	(173 – 193)
**$VO2_{peak}$/kg**	38,7	(33,9 – 45,2)	35,0	(30,0 – 40,9)
**VO_2 as % of predicted**	74,7	(61,4 – 90)	81	(67,4 – 97,4)
**W as % of predicted**	80,3	(65,5 -92 ,7)	86,3	(67,2 – 97,4)
**HR as % of predicted**	95,3	(89,5 – 99,6)	96,7	(91,0 – 101,6)
**VE/VCO_2 Slope**	27,4	(24,1 –32,0)	25,4	(23,2 – 29,0)

Supplemental figure 5: reference values for $VO2_{peak}$, W_{peak} and $O2pulse_{peak}$ and median HR_{peak}, VO_{2peak}/kg, VO_2 as% of predicted, Wpeak as % of predicted and HR as % of predicted patients with ASD, in males and females separately.

ASD

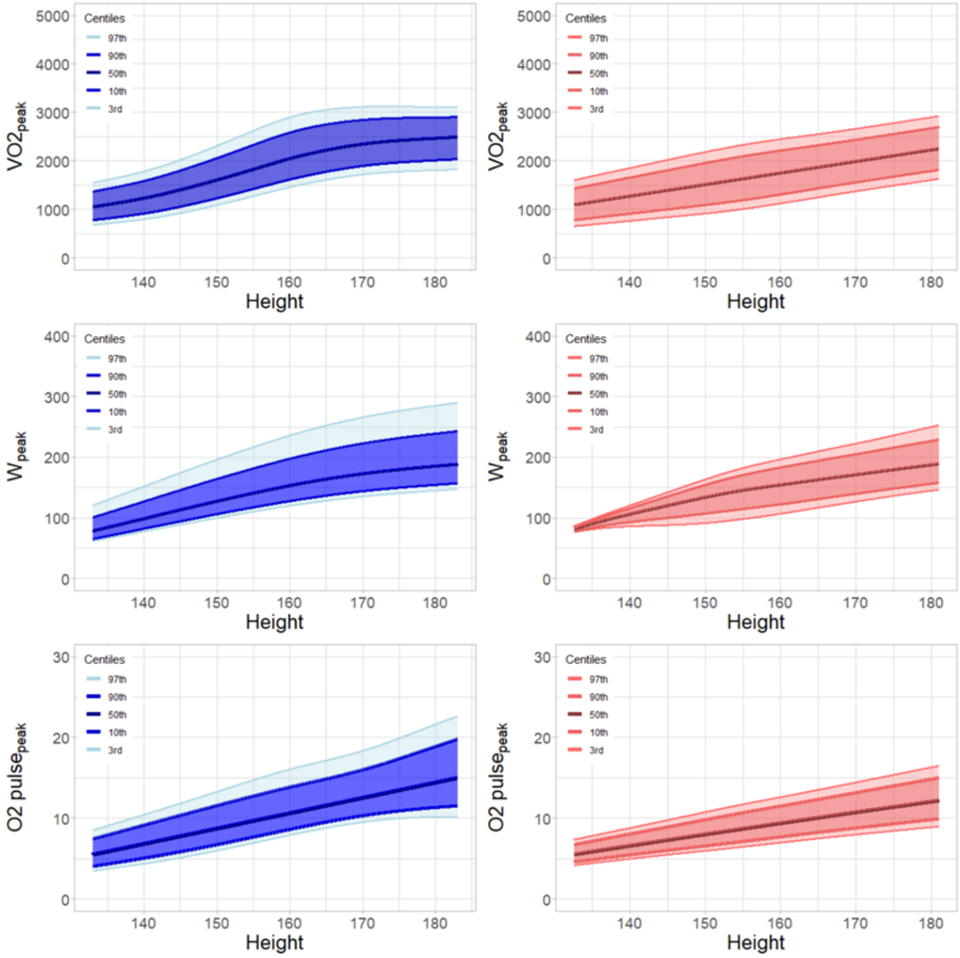

	Male		Female	
	Median	(25th – 75th percentile)	Median	(25th – 75th percentile)
**HR peak**	187	(178 – 195)	188	(178 – 194)
**$VO2_{peak}$ /kg**	39,9	(36,4 – 45,5)	34,6	(27,9 – 41,6)
**VO_2 as % of predicted**	85,2	(68,1 – 92,3)	83,8	(70,9 – 93,4)
**W as % of predicted**	84,9	(69,5 – 95,8)	90,1	(77,8 – 100,2)
**HR as % of predicted**	99,9	(94,1 – 103,3)	98,7	(94,1 – 102,1)
**VE/VCO_2 Slope**	30,2	(26,6 – 32,3)	29,0	(25,9 – 32,3)

Supplemental figure 6: reference values for VO_{2peak}, W_{peak} and $O2pulse_{peak}$ and median HR_{peak}, VO_{2peak}/kg, VO_2 as% of predicted, Wpeak as % of predicted and HR as % of predicted in patients with AV valve abnormalities, in males and females separately.

AV valve abnormality

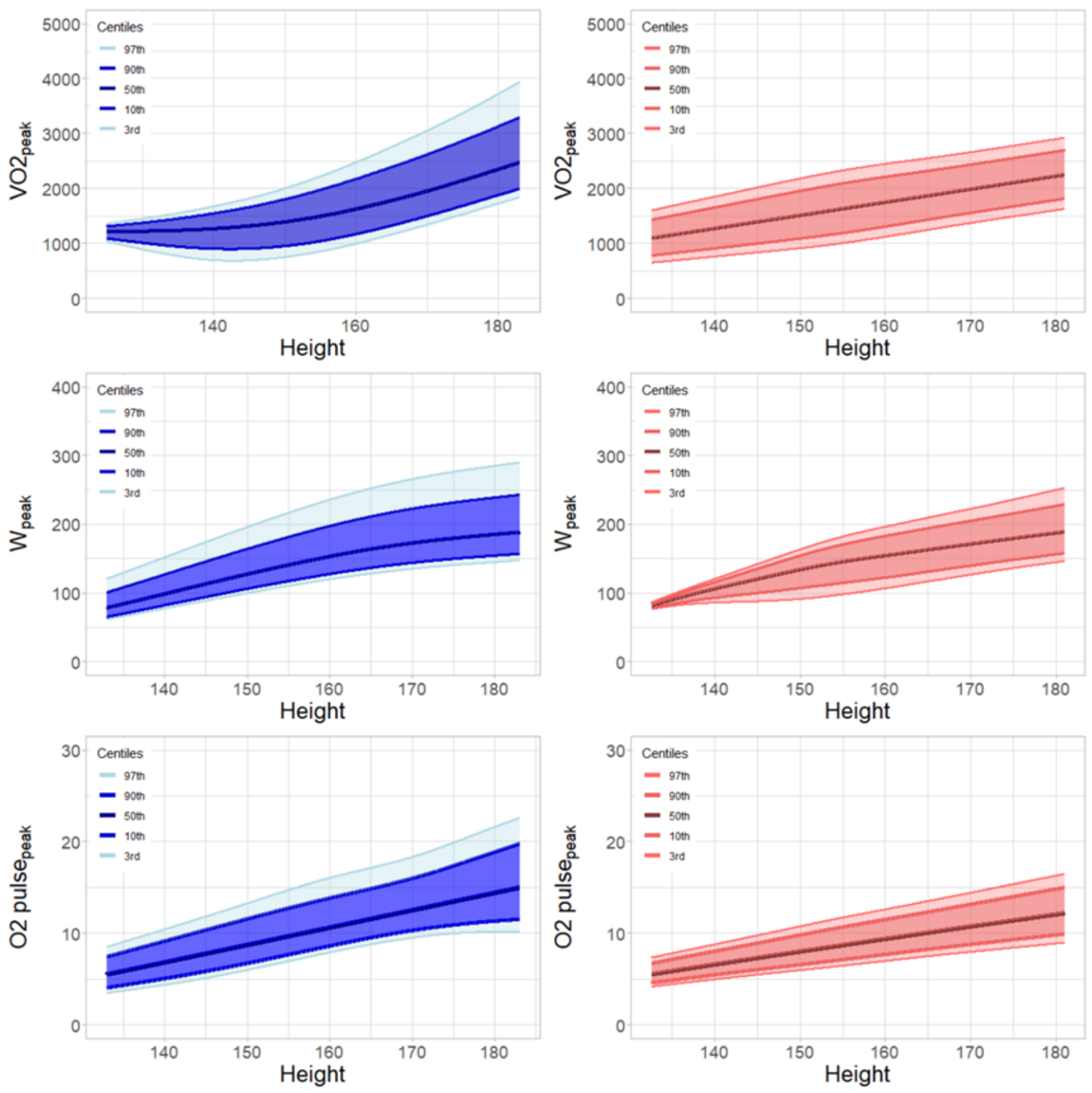

	Male		Female	
	Median	(25th – 75th percentile)	Median	(25th – 75th percentile)
**HR peak**	187	(178 – 195)	188	(178 – 194)
**$VO2_{peak}$/kg**	39,9	(36,4 – 45,5)	34,6	(27,9 – 41,6)
**VO_2 as % of predicted**	85,2	(68,1 – 92,3)	83,8	(70,9 – 93,4)
**W as % of predicted**	84,9	(69,5 – 95,8)	90,1	(77,8 – 100,2)
**HR as % of predicted**	99,9	(94,1 – 103,3)	98,7	(94,1 – 102,1)
**VE/VCO_2 Slope**	33,2	(29,8 – 43,7)	28,0	(24,9 – 34,3)

Supplemental figure 7: reference values for VO_{2peak}, W_{peak} and $O2pulse_{peak}$ and median HR_{peak}, VO_{2peak}/kg, VO_2 as% of predicted, Wpeak as % of predicted and HR as % of predicted in patients with pulmonary stenosis, in males and females separately.

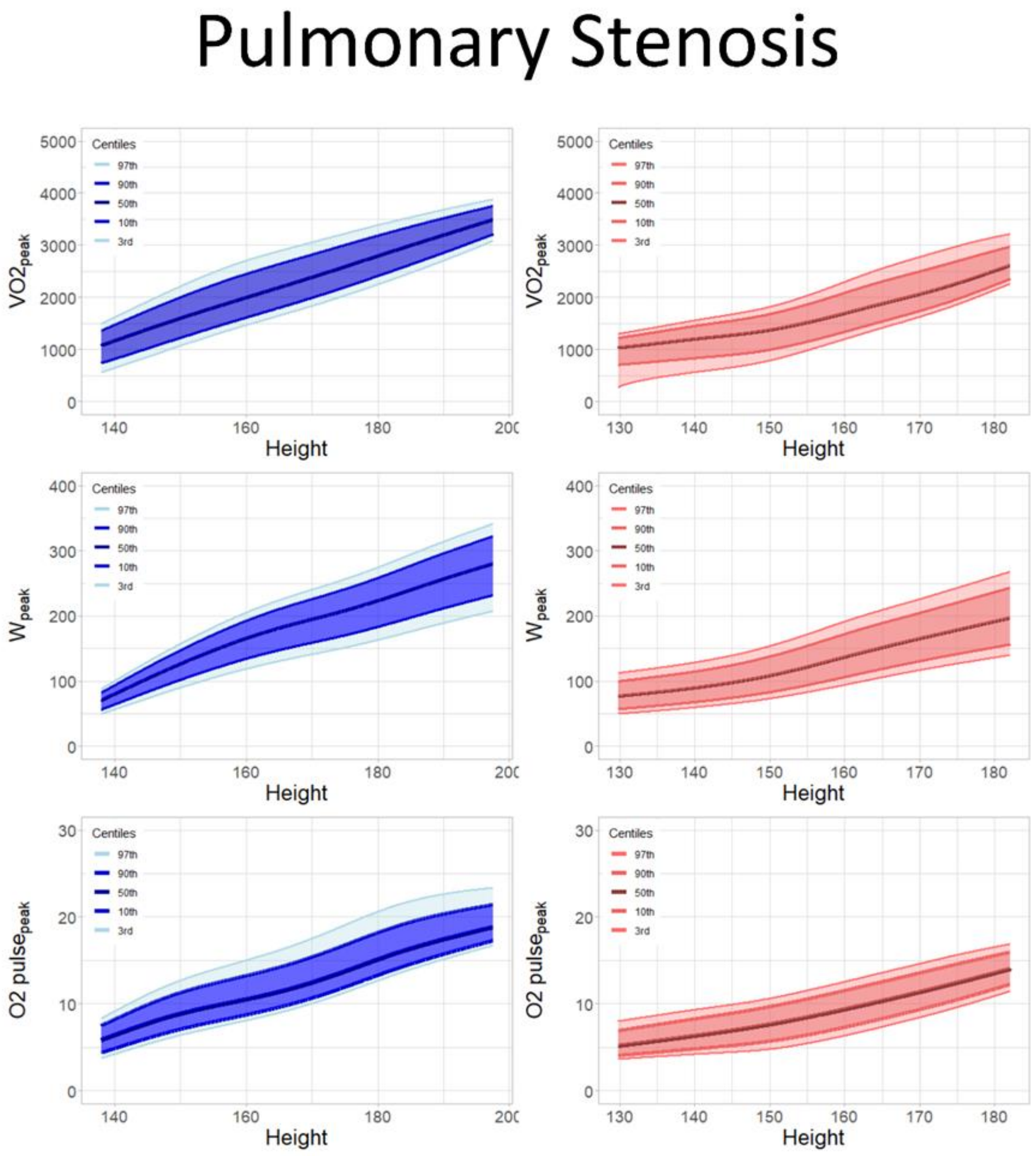

	Male		Female	
	Median	(25th – 75th percentile)	Median	(25th – 75th percentile)
**HR peak**	183	(171 – 190)	184	(174 – 193)
**$VO2_{peak}$ /kg**	42,9	(38,3 -47,0)	34,0	(28,5 – 39,1)
**VO_2 as % of predicted**	79,8	(70,4 – 89,1)	84,1	(70,5 – 90,4)
**W as % of predicted**	79,1	(73,3 – 89,4)	78,3	(67,0 – 89,4)
**HR as % of predicted**	97,1	(89,9 – 99,7)	97,0	(94,1 – 102,1)
**VE/VCO_2 Slope**	29,9	(25,5 – 32,6)	31,3	(28,8 – 34,9)

Supplemental figure 8: reference values for VO_{2peak}, W_{peak} and $O2pulse_{peak}$ and median HR_{peak}, VO_{2peak}/kg, VO_2 as% of predicted, Wpeak as % of predicted and HR as % of in patients with Aortic Coartation, in males and females separately.

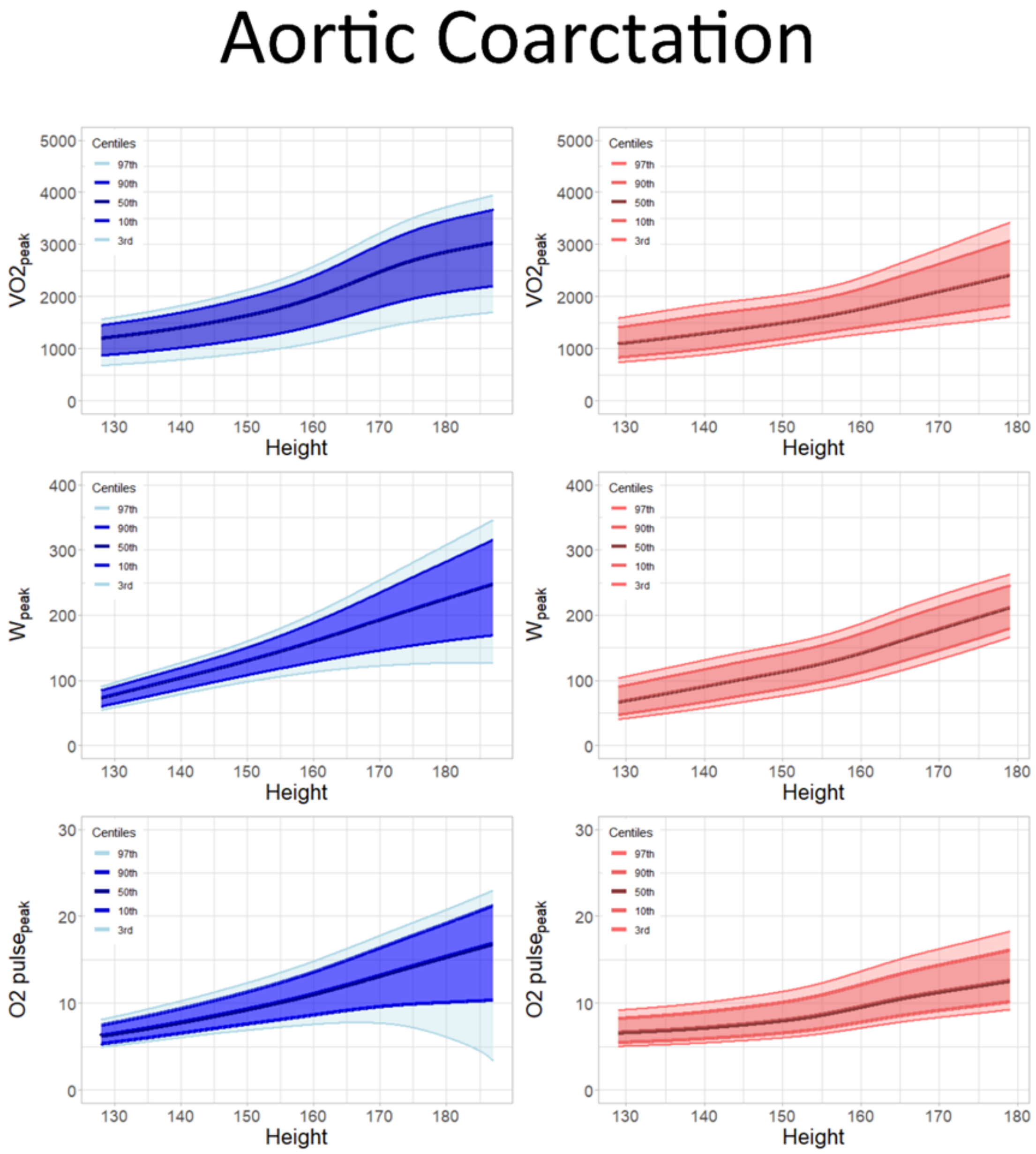

	Male		Female	
	**Median**	**(25th – 75th percentile)**	**Median**	**(25th – 75th percentile)**
**HR peak**	182	(173 – 190)	184	(176 – 190)
**$VO2_{peak}$ /kg**	43,4	(37,4 – 48,1)	35,4	(31,8 – 40,2)
**VO_2 as % of predicted**	81,0	(70,7 – 97,5)	86,1	(72,4 – 97,0)
**W as % of predicted**	84,1	(69,7 – 97,7)	83,0	(69,7 – 95,8)
**HR as % of predicted**	96,6	(90,6 – 100,6)	96,7	(92,8 – 100,03)
**VE/VCO_2 Slope**	29,5	(23,1 – 34,9)	29,6	(23,4 – 35,1)

Supplemental table 2: Tukey analysis of VO_{2peak}

	diff	lwr	upr	p adj
Univentricular Heart vs				
ASD	430.332	177.516	683.149	**<0.001**
VSD	361.032	175.242	546.821	**<0.001**
AV valve abnormality	180.022	-62.841	422.884	0.341
Pulmonary stenosis	528.066	287.436	768.696	**<0.001**
Aortic stenosis	607.225	423.330	791.120	**<0.001**
Aortic Coarctation	521.064	321.835	720.293	**<0.001**
Transposition of the great arteries	513.125	327.890	698.360	**<0.001**
Tetralogy of Fallot	358.432	187.632	529.232	**<0.001**
AV valve abnormality vs				
ASD	250.311	-51.813	552.435	0.199
VSD	181.010	-67.748	429.768	0.367
Pulmonary stenosis	348.044	56.042	640.047	0.007
Aortic stenosis	427.203	179.857	674.549	**<0.001**
Aortic Coarctation	341.043	82.093	599.993	**0.001**
Transposition of the great arteries	333.103	84.759	581.448	**0.001**
Tetralogy of Fallot	178.411	-59.361	416.182	0.324
Tetralogy of Fallot vs				
ASD	71.900	-176.030	319.830	0.993
VSD	2.600	-176.484	181.683	1.000
Pulmonary stenosis	169.634	-65.857	405.125	0.382
Aortic stenosis	248.793	71.675	425.910	**<0.001**
Aortic Coarctation	162.632	-30.359	355.623	0.180
Transposition of the great arteries	154.693	-23.816	333.202	0.151
VSD vs				
ASD	69.301	-189.184	327.786	0.996
Pulmonary stenosis	167.034	-79.545	413.613	0.471
Aortic stenosis	246.193	54.579	437.807	0.002
Aortic Coarctation	160.033	-46.342	366.408	0.280
Transposition of the great arteries	152.093	-40.807	344.994	0.258
ASD vs				
Pulmonary stenosis	97.733	-202.599	398.066	0.985
Aortic stenosis	176.892	-80.234	434.019	0.448
Aortic Coarctation	90.732	-177.576	359.040	0.981
Transposition of the great arteries	82.793	-175.294	340.880	0.986
Transposition of the great arteries vs				
Pulmonary stenosis	14.941	-231.221	261.102	1.000
Aortic stenosis	94.100	-96.977	285.176	0.841
Aortic Coarctation	7.939	-197.937	213.816	1.000
Aortic Coarctation vs				
Pulmonary stenosis	7.001	-249.856	263.859	1.000
Aortic stenosis	86.160	-118.511	290.832	0.929
Pulmonary stenosis vs				
Aortic stenosis	79.159	-165.996	324.314	0.986

Supplemental table 3: Tukey analysis of VO_{2peak}/kg

	diff	lwr	upr	p adj
Univentricular Heart vs				
ASD	5.619	-0.751	11.989	0.134
VSD	7.053	2.608	11.498	**<0.001**
AV valve abnormality	0.751	-5.520	7.023	1.000
Pulmonary stenosis	8.914	2.068	15.760	0.002
Aortic stenosis	9.450	4.933	13.966	**<0.001**
Aortic Coarctation	10.501	5.297	15.704	**<0.001**
Transposition of the great arteries	9.290	4.518	14.062	**<0.001**
Tetralogy of Fallot	6.010	1.663	10.357	0.001
AV valve abnormality vs				
ASD	5.619	-0.751	11.989	0.134
VSD	7.053	2.608	11.498	**<0.001**
Pulmonary stenosis	9.665	1.619	17.712	0.006
Aortic stenosis	10.201	4.014	16.388	**<0.001**
Aortic Coarctation	11.252	4.547	17.957	**<0.001**
Transposition of the great arteries	10.041	3.665	16.417	**<0.001**
Tetralogy of Fallot	6.761	0.697	12.826	0.016
Tetralogy of Fallot vs				
ASD	0.391	-5.775	6.557	1.000
VSD	1.043	-3.105	5.191	0.997
Pulmonary stenosis	2.904	-3.752	9.560	0.912
Aortic stenosis	3.440	-0.785	7.664	0.217
Aortic Coarctation	4.882	-1.915	11.678	0.382
Transposition of the great arteries	3.280	-1.217	7.776	0.360
VSD vs				
ASD	1.434	-4.802	7.670	0.999
Pulmonary stenosis	1.861	-4.860	8.582	0.995
Aortic stenosis	2.396	-1.928	6.721	0.729
Aortic Coarctation	3.448	-1.590	8.485	0.452
Transposition of the great arteries	2.237	-2.354	6.828	0.847
ASD vs				
Pulmonary stenosis	3.295	-4.828	11.418	0.941
Aortic stenosis	3.830	-2.456	10.117	0.615
Aortic Coarctation	4.882	-1.915	11.678	0.382
Transposition of the great arteries	3.671	-2.802	10.143	0.704
Transposition of the great arteries vs				
Pulmonary stenosis	0.376	-6.565	7.317	1.000
Aortic stenosis	0.160	-4.500	4.819	1.000
Aortic Coarctation	1.211	-4.118	6.539	0.999
Aortic Coarctation vs				
Pulmonary stenosis	1.587	-5.658	8.831	0.999
Aortic stenosis	1.051	-4.049	6.152	0.999
Pulmonary stenosis vs				
Aortic stenosis	0.536	-6.232	7.303	1.000

Supplemental table 4: Tukey analysis of W_{peak}

	diff	lwr	upr	p adj
Univentricular Heart vs				
ASD	5.619	-0.751	11.989	0.134
VSD	7.053	2.608	11.498	**<0.001**
AV valve abnormality	0.751	-5.520	7.023	1.000
Pulmonary stenosis	8.914	2.068	15.760	0.002
Aortic stenosis	9.450	4.933	13.966	**<0.001**
Aortic Coarctation	10.501	5.297	15.704	**<0.001**
Transposition of the great arteries	9.290	4.518	14.062	**<0.001**
Tetralogy of Fallot	6.010	1.663	10.357	0.001
AV valve abnormality vs				
ASD	6.370	-1.275	14.016	0.191
VSD	7.804	1.669	13.940	0.003
Pulmonary stenosis	9.665	1.619	17.712	0.006
Aortic stenosis	10.201	4.014	16.388	**<0.001**
Aortic Coarctation	11.252	4.547	17.957	**<0.001**
Transposition of the great arteries	10.041	3.665	16.417	**<0.001**
Tetralogy of Fallot	6.761	0.697	12.826	0.016
Tetralogy of Fallot vs				
ASD	0.391	-5.775	6.557	1.000
VSD	1.043	-3.105	5.191	0.997
Pulmonary stenosis	2.904	-3.752	9.560	0.912
Aortic stenosis	3.440	-0.785	7.664	0.217
Aortic Coarctation	4.882	-1.915	11.678	0.382
Transposition of the great arteries	3.280	-1.217	7.776	0.360
VSD vs				
ASD	1.434	-4.802	7.670	0.999
Pulmonary stenosis	1.861	-4.860	8.582	0.995
Aortic stenosis	2.396	-1.928	6.721	0.729
Aortic Coarctation	3.448	-1.590	8.485	0.452
Transposition of the great arteries	2.237	-2.354	6.828	0.847
ASD vs				
Pulmonary stenosis	3.295	-4.828	11.418	0.941
Aortic stenosis	3.830	-2.456	10.117	0.615
Aortic Coarctation	4.882	-1.915	11.678	0.382
Transposition of the great arteries	3.671	-2.802	10.143	0.704
Transposition of the great arteries vs				
Pulmonary stenosis	0.376	-6.565	7.317	1.000
Aortic stenosis	0.160	-4.500	4.819	1.000
Aortic Coarctation	1.211	-4.118	6.539	0.999
Aortic Coarctation vs				
Pulmonary stenosis	1.587	-5.658	8.831	0.999
Aortic stenosis	1.051	-4.049	6.152	0.999
Pulmonary stenosis vs				
Aortic stenosis	0.536	-6.232	7.303	1.000

Supplemental table 5: Tukey analysis of W_{peak} /kg

	diff	lwr	upr	p adj
Univentricular Heart vs				
ASD	0.538	0.245	0.831	**<0.001**
VSD	0.567	0.352	0.783	**<0.001**
AV valve abnormality	0.321	0.040	0.603	0.012
Pulmonary stenosis	0.518	0.239	0.797	**<0.001**
Aortic stenosis	0.719	0.506	0.933	**<0.001**
Aortic Coarctation	0.574	0.343	0.805	**<0.001**
Transposition of the great arteries	0.677	0.463	0.892	**<0.001**
Tetralogy of Fallot	0.500	0.302	0.698	**<0.001**
AV valve abnormality vs				
ASD	0.217	-0.134	0.567	0.599
VSD	0.067	-0.140	0.275	0.985
Pulmonary stenosis	0.196	-0.142	0.535	0.681
Aortic stenosis	0.220	0.014	0.425	0.026
Aortic Coarctation	0.075	-0.149	0.299	0.982
Transposition of the great arteries	0.178	-0.029	0.385	0.160
Tetralogy of Fallot	0.178	-0.098	0.454	0.538
Tetralogy of Fallot vs				
ASD	0.391	-5.775	6.557	1.000
VSD	1.043	-3.105	5.191	0.997
Pulmonary stenosis	2.904	-3.752	9.560	0.912
Aortic stenosis	3.440	-0.785	7.664	0.217
Aortic Coarctation	4.882	-1.915	11.678	0.382
Transposition of the great arteries	3.280	-1.217	7.776	0.360
VSD vs				
ASD	0.029	-0.271	0.329	1.000
Pulmonary stenosis	0.049	-0.237	0.335	1.000
Aortic stenosis	0.152	-0.070	0.375	0.454
Aortic Coarctation	0.007	-0.232	0.247	1.000
Transposition of the great arteries	0.110	-0.113	0.334	0.840
ASD vs				
Pulmonary stenosis	0.020	-0.328	0.369	1.000
Aortic stenosis	0.181	-0.117	0.479	0.622
Aortic Coarctation	0.036	-0.275	0.347	1.000
Transposition of the great arteries	0.139	-0.160	0.439	0.880
Transposition of the great arteries vs				
Pulmonary stenosis	0.160	-0.126	0.445	0.724
Aortic stenosis	0.042	-0.180	0.264	1.000
Aortic Coarctation	0.103	-0.136	0.342	0.919
Aortic Coarctation vs				
Pulmonary stenosis	0.056	-0.241	0.354	1.000
Aortic stenosis	0.145	-0.092	0.382	0.615
Pulmonary stenosis vs				
Aortic stenosis	0.202	-0.083	0.486	0.405

Supplemental table 6: Tukey analysis of HR_{peak}

	diff	lwr	upr	p adj
Univentricular Heart vs				
ASD	13.199	6.195	20.203	**<0.001**
VSD	8.457	3.305	13.609	**<0.001**
AV valve abnormality	10.484	3.723	17.244	**<0.001**
Pulmonary stenosis	10.134	3.437	16.832	**<0.001**
Aortic stenosis	13.603	8.482	18.725	**<0.001**
Aortic Coarctation	8.913	3.378	14.448	**<0.001**
Transposition of the great arteries	8.207	3.070	13.344	**<0.001**
Tetralogy of Fallot	6.559	1.817	11.302	0.001
AV valve abnormality vs				
ASD	2.716	-5.670	11.101	0.985
VSD	2.026	-4.888	8.941	0.992
Pulmonary stenosis	0.196	-0.142	0.535	0.681
Aortic stenosis	3.120	-3.772	10.012	0.896
Aortic Coarctation	0.349	-7.782	8.481	1.000
Transposition of the great arteries	2.277	-4.626	9.180	0.984
Tetralogy of Fallot	3.924	-2.691	10.539	0.653
Tetralogy of Fallot vs				
ASD	6.640	-0.223	13.503	0.067
VSD	1.898	-3.062	6.857	0.959
Pulmonary stenosis	3.575	-2.976	10.125	0.749
Aortic stenosis	7.044	2.116	11.972	**<0.001**
Aortic Coarctation	2.354	-3.002	7.710	0.911
Transposition of the great arteries	1.647	-3.296	6.591	0.982
VSD vs				
ASD	4.742	-2.410	11.894	0.502
Pulmonary stenosis	1.677	-5.176	8.530	0.998
Aortic stenosis	5.146	-0.177	10.469	0.067
Aortic Coarctation	0.456	-5.266	6.178	1.000
Transposition of the great arteries	0.250	-5.087	5.588	1.000
ASD vs				
Pulmonary stenosis	3.065	-5.270	11.400	0.968
Aortic stenosis	0.404	-6.726	7.535	1.000
Aortic Coarctation	4.286	-3.147	11.719	0.688
Transposition of the great arteries	4.992	-2.149	12.134	0.425
Transposition of the great arteries vs				
Pulmonary stenosis	1.927	-4.914	8.769	0.994
Aortic stenosis	5.397	0.088	10.705	0.043
Aortic Coarctation	0.707	-5.002	6.415	1.000
Aortic Coarctation vs				
Pulmonary stenosis	1.221	-5.924	8.366	1.000
Aortic stenosis	4.690	-1.005	10.385	0.205
Pulmonary stenosis vs				
Aortic stenosis	3.469	-3.361	10.299	0.817

Supplemental table 7: Tukey analysis of VO_{2peak} as % of predicted

	diff	lwr	upr	p adj
Univentricular Heart vs				
ASD	19.354	11.002	27.706	**<0.001**
VSD	16.446	10.308	22.584	**<0.001**
AV valve abnormality	7.539	-0.485	15.562	0.085
Pulmonary stenosis	16.491	8.542	24.441	**<0.001**
Aortic stenosis	21.995	15.919	28.070	**<0.001**
Aortic Coarctation	20.485	13.904	27.067	**<0.001**
Transposition of the great arteries	17.053	10.933	23.172	**<0.001**
Tetralogy of Fallot	12.549	6.907	18.192	**<0.001**
AV valve abnormality vs				
ASD	11.816	1.835	21.797	0.008
VSD	8.908	0.690	17.125	0.022
Pulmonary stenosis	8.953	-0.694	18.599	0.094
Aortic stenosis	14.456	6.285	22.627	**<0.001**
Aortic Coarctation	12.947	4.392	21.501	**<0.001**
Transposition of the great arteries	9.514	1.310	17.718	0.010
Tetralogy of Fallot	5.011	-2.844	12.866	0.556
Tetralogy of Fallot vs				
ASD	6.805	-1.386	14.995	0.195
VSD	3.897	-2.019	9.813	0.511
Pulmonary stenosis	3.942	-3.838	11.721	0.819
Aortic stenosis	9.445	3.594	15.296	**<0.001**
Aortic Coarctation	7.936	1.560	14.311	0.004
Transposition of the great arteries	4.503	-1.394	10.400	0.300
VSD vs				
ASD	2.908	-5.631	11.447	0.980
Pulmonary stenosis	0.045	-8.101	8.191	1.000
Aortic stenosis	5.548	-0.782	11.878	0.141
Aortic Coarctation	4.039	-2.779	10.857	0.655
Transposition of the great arteries	0.607	-5.766	6.979	1.000
ASD vs				
Pulmonary stenosis	2.863	-7.058	12.785	0.993
Aortic stenosis	2.640	-5.854	11.135	0.989
Aortic Coarctation	1.131	-7.733	9.995	1.000
Transposition of the great arteries	2.302	-6.224	10.828	0.996
Transposition of the great arteries vs				
Pulmonary stenosis	0.562	-7.570	8.694	1.000
Aortic stenosis	4.942	-1.371	11.254	0.267
Aortic Coarctation	3.433	-3.369	10.234	0.822
Aortic Coarctation vs				
Pulmonary stenosis	3.994	-4.491	12.480	0.873
Aortic stenosis	1.509	-5.252	8.271	0.999
Pulmonary stenosis vs				
Aortic stenosis	5.503	-2.595	13.602	0.466

Supplemental table 8: Tukey analysis of W_{peak} as % of predicted

	diff	lwr	upr	p adj
Univentricular Heart vs				
ASD	23.574	15.412	31.736	**<0.001**
VSD	20.671	14.673	26.669	**<0.001**
AV valve abnormality	9.639	1.799	17.480	0.004
Pulmonary stenosis	16.536	8.768	24.305	**<0.001**
Aortic stenosis	22.785	16.849	28.722	**<0.001**
Aortic Coarctation	20.672	14.240	27.104	**<0.001**
Transposition of the great arteries	21.004	15.024	26.984	**<0.001**
Tetralogy of Fallot	15.201	9.687	20.715	**<0.001**
AV valve abnormality vs				
ASD	13.934	4.180	23.688	**<0.001**
VSD	11.031	3.001	19.062	0.001
Pulmonary stenosis	6.897	-2.530	16.324	0.359
Aortic stenosis	13.146	5.161	21.131	**<0.001**
Aortic Coarctation	11.033	2.673	19.393	0.001
Transposition of the great arteries	11.365	3.347	19.382	**<0.001**
Tetralogy of Fallot	5.561	-2.115	13.238	0.373
Tetralogy of Fallot vs				
ASD	8.373	0.369	16.377	0.032
VSD	5.470	-0.311	11.252	0.080
Pulmonary stenosis	1.335	-6.267	8.938	1.000
Aortic stenosis	7.585	1.866	13.303	0.001
Aortic Coarctation	5.472	-0.759	11.702	0.139
Transposition of the great arteries	5.803	0.040	11.566	0.047
VSD vs				
ASD	2.903	-5.442	11.248	0.977
Pulmonary stenosis	4.135	-3.826	12.095	0.797
Aortic stenosis	2.114	-4.072	8.301	0.979
Aortic Coarctation	0.001	-6.661	6.664	1.000
Transposition of the great arteries	0.333	-5.894	6.561	1.000
ASD vs				
Pulmonary stenosis	7.038	-2.658	16.733	0.371
Aortic stenosis	0.788	-7.513	9.089	1.000
Aortic Coarctation	2.901	-5.761	11.563	0.982
Transposition of the great arteries	2.569	-5.763	10.902	0.989
Transposition of the great arteries vs				
Pulmonary stenosis	4.468	-3.479	12.415	0.717
Aortic stenosis	1.781	-4.387	7.950	0.993
Aortic Coarctation	0.332	-6.315	6.978	1.000
Aortic Coarctation vs				
Pulmonary stenosis	4.136	-4.156	12.429	0.832
Aortic stenosis	1.509	-5.252	8.271	0.999
Pulmonary stenosis vs				
Aortic stenosis	6.249	-1.665	14.164	0.256

Supplemental table 9: Tukey analysis of HR_{peak} as % of predicted

	diff	lwr	upr	p adj
Univentricular Heart vs				
ASD	6.992	3.312	10.672	**<0.001**
VSD	4.485	1.778	7.192	**<0.001**
AV valve abnormality	5.453	1.901	9.006	**<0.001**
Pulmonary stenosis	5.160	1.640	8.679	**<0.001**
Aortic stenosis	7.111	4.420	9.802	**<0.001**
Aortic Coarctation	4.641	1.732	7.549	**<0.001**
Transposition of the great arteries	4.211	1.512	6.910	**<0.001**
Tetralogy of Fallot	3.399	0.907	5.891	0.001
AV valve abnormality vs				
ASD	1.539	-2.868	5.945	0.976
VSD	0.968	-2.665	4.602	0.996
Pulmonary stenosis	0.294	-3.979	4.567	1.000
Aortic stenosis	1.658	-1.964	5.279	0.889
Aortic Coarctation	0.812	-2.973	4.598	0.999
Transposition of the great arteries	1.242	-2.385	4.870	0.979
Tetralogy of Fallot	2.054	-1.421	5.530	0.658
Tetralogy of Fallot vs				
ASD	3.593	-0.013	7.200	0.052
VSD	1.086	-1.520	3.692	0.933
Pulmonary stenosis	1.761	-1.681	5.203	0.811
Aortic stenosis	3.712	1.123	6.302	**<0.001**
Aortic Coarctation	1.242	-1.572	4.057	0.909
Transposition of the great arteries	0.812	-1.786	3.410	0.988
VSD vs				
ASD	2.507	-1.251	6.265	0.493
Pulmonary stenosis	0.675	-2.926	4.276	1.000
Aortic stenosis	2.626	-0.171	5.423	0.086
Aortic Coarctation	0.156	-2.851	3.163	1.000
Transposition of the great arteries	0.274	-2.531	3.079	1.000
ASD vs				
Pulmonary stenosis	1.832	-2.547	6.212	0.932
Aortic stenosis	0.119	-3.628	3.866	1.000
Aortic Coarctation	2.351	-1.555	6.257	0.635
Transposition of the great arteries	2.781	-0.971	6.534	0.341
Transposition of the great arteries vs				
Pulmonary stenosis	0.949	-2.646	4.544	0.996
Aortic stenosis	2.900	0.111	5.689	0.034
Aortic Coarctation	0.430	-2.569	3.429	1.000
Aortic Coarctation vs				
Pulmonary stenosis	0.519	-3.236	4.273	1.000
Aortic stenosis	2.470	-0.522	5.462	0.203
Pulmonary stenosis vs				
Aortic stenosis	1.951	-1.638	5.540	0.753

Supplemental table 10: Tukey analysis of O_2 pulse$_{peak}$

	diff	lwr	upr	p adj
Univentricular Heart vs				
ASD	1.728	0.319	3.138	0.005
VSD	1.537	0.501	2.573	**<0.001**
AV valve abnormality	0.565	-0.795	1.925	0.934
Pulmonary stenosis	2.359	1.012	3.707	**<0.001**
Aortic stenosis	2.627	1.600	3.654	**<0.001**
Aortic Coarctation	2.533	1.422	3.644	**<0.001**
Transposition of the great arteries	2.384	1.351	3.417	**<0.001**
Tetralogy of Fallot	3.399	0.907	5.891	0.001
AV valve abnormality vs				
ASD	1.163	-0.525	2.851	0.446
VSD	0.968	-2.665	4.602	0.996
Pulmonary stenosis	1.794	0.157	3.431	0.020
Aortic stenosis	2.062	0.676	3.447	**<0.001**
Aortic Coarctation	1.968	0.520	3.417	0.001
Transposition of the great arteries	1.819	0.429	3.209	0.002
Tetralogy of Fallot	0.952	-0.380	2.284	0.393
Tetralogy of Fallot vs				
ASD	0.211	-1.170	1.593	1.000
VSD	0.020	-0.978	1.018	<0.001
Pulmonary stenosis	0.842	-0.477	2.161	0.555
Aortic stenosis	1.110	0.121	2.099	0.015
Aortic Coarctation	1.016	-0.060	2.092	0.082
Transposition of the great arteries	0.867	-0.128	1.863	0.146
VSD vs				
ASD	0.191	-1.249	1.631	1.000
Pulmonary stenosis	0.822	-0.558	2.202	0.648
Aortic stenosis	1.090	0.021	2.159	0.042
Aortic Coarctation	0.996	-0.153	2.146	0.151
Transposition of the great arteries	0.847	-0.227	1.922	0.258
ASD vs				
Pulmonary stenosis	0.631	-1.047	2.309	0.963
Aortic stenosis	0.899	-0.535	2.332	0.581
Aortic Coarctation	0.805	-0.690	2.300	0.763
Transposition of the great arteries	0.656	-0.782	2.094	0.891
Transposition of the great arteries vs				
Pulmonary stenosis	0.025	-1.352	1.402	<0.001
Aortic stenosis	0.243	-0.823	1.308	0.999
Aortic Coarctation	0.149	-0.998	1.296	1.000
Aortic Coarctation vs				
Pulmonary stenosis	0.174	-1.263	1.611	1.000
Aortic stenosis	0.094	-1.048	1.235	1.000
Pulmonary stenosis vs				
Aortic stenosis	0.268	-1.105	1.641	1.000
